# MECOM and the PRDM gene family in uterine endometrial cancer: bioinformatics and experimental insights into pathogenesis and therapeutic potentials

**DOI:** 10.1186/s10020-024-00946-0

**Published:** 2024-10-28

**Authors:** Meng Lou, Lian Zou, Liying Zhang, Yongquan Lu, Jia Chen, Beige Zong

**Affiliations:** 1 Department of Obstetrics and Gynecology, Chongqing Emergency Medical Center, Chongging University Central Hospital, Chongqing, 400000 China; 2 Department of Obstetrics and Gynecology, The Second Affiliated Hospital of Guangxi Medical University, Nanning, 530000 China; 3 Department of Clinical Laboratory, Chongqing Emergency Medical Center, Chongging University Central Hospital, Chongqing, 400000 China; 4 Department of General Surgery, Chongqing Emergency Medical Center, Chongging University Central Hospital, No.1 Jiankang Road, Yuzhong District, Chongqing, 400000 China; 5 Department of Obstetrics and Gynecology, Xinqiao Hospital, Army Medical University, Chongqing, 400037 China

**Keywords:** PRDM gene family, Uterine endometrial cancer, MECOM, Expression patterns, Methylation status, TGF-beta signaling pathway

## Abstract

To elucidate the expression profiles, methylation states, and clinicopathological significance of the PRDM gene family, focusing on the MECOM gene's role in uterine endometrial cancer (UCEC) and its molecular interactions with the TGF-beta signaling pathway. Our methodology combined detailed bioinformatics analyses using UALCAN and GEPIA with in vitro assessments in HEC-1-A cells. Techniques included CRISPR-Cas9 for gene editing and various cellular assays (CCK-8, flow cytometry, Transwell) to evaluate the effects of MECOM on cell proliferation, migration, and apoptosis, alongside Western blot analysis for protein regulation in the TGF-beta pathway. MECOM was upregulated in UCEC tissues, influencing tumor cell behavior significantly. Knockout studies demonstrated reduced proliferation and migration and increased apoptosis, while overexpression showed reverse effects. Mechanistically, MECOM modulated critical proteins within the TGF-beta pathway, impacting cell cycle dynamics and apoptotic processes. The PRDM gene family, particularly MECOM, plays a crucial role in the pathogenesis and progression of UCEC, suggesting its utility as a target for novel therapeutic interventions. Our findings offer valuable insights for future research and potential clinical application in managing uterine endometrial cancer.

**Supplementary Information:**

The online version contains supplementary material available at 10.1186/s10020-024-00946-0.

## Introduction

The incidence of uterine corpus endometrial carcinoma (UCEC), a type of uterine cancer, has been steadily increasing at a rate of approximately 1.3% per year from 2007 to 2016 (Lortet-Tieulent et al. 2018; Pfeiffer et al., 2018; Lheureux et al. 2018; Chiofalo et al. 2020). This rise in incidence can be attributed, in part, to the decreasing fertility rates and the increasing prevalence of obesity (Lortet-Tieulent et al. 2018; Pfeiffer et al., 2018; Lheureux et al. 2018; Chiofalo et al. 2020). Despite significant efforts in diagnosing and treating UCEC, the mortality rate of this cancer has been on the rise over the past decade (Siegel et al. 2020; Oaknin et al. 2022; Kato et al. 2021; Gao et al. 2022). The management of UCEC is challenging due to its unpredictable clinical behavior and the lack of well-defined molecular therapeutic targets (Liu et al. 2014; Crosbie et al. 2022; van den Heerik et al. 2021; Karpel et al. 2023). Despite significant advancements in understanding the molecular mechanisms of tumorigenesis and development (Liu et al. 2018; Suryo Rahmanto et al., 2020) and in targeted molecular therapy research (Morice et al. 2016; Urick and Bell 2019), molecular profiling has been employed for many years to manage these advanced and recurrent UCEC patients. For example, progesterone receptor and estrogen receptor status are used to evaluate whether patients can receive hormone therapy. However, even among some patients with progesterone receptor and estrogen receptor positivity, single-agent aromatase inhibitors only achieve a 10% response rate (Crosbie et al. 2022). Therefore, identifying new targets and further understanding the molecular biology of UCEC remains a pressing priority. These efforts will aid in developing effective targeted chemotherapy strategies in the future.

MECOM and PRDM family members play multifaceted roles in cancer. PRDM stands for PR Domain-Containing Protein, and MECOM stands for MDS1 and EVI1 Complex Locus Protein. The MECOM gene is also known as PRDM3, one of its aliases (Li et al. 2022). These proteins influence tumor initiation, progression, and metastasis by regulating gene expression, cell proliferation, differentiation, apoptosis, and signal transduction (Nishikawa et al. 2007; Chittka et al. 2012; Deng and Huang 2004). The PRDM family affects gene expression through chromatin modifications mediated by their PR domains, which contain the SET domain typical of the protein family (Mistik and Sayar 2022). The PRDM genes have been implicated in cell proliferation and differentiation (Nishikawa et al. 2007; Chittka et al. 2012), apoptosis (Deng and Huang 2004), cell cycle progression (Deng and Huang 2004), inflammation (Savage et al. 2017), metabolism (Mzoughi et al. 2020), and the immune microenvironment (Casamassimi et al., 2020). Recent studies have suggested that dysregulation of PRDM gene expression in various malignant tumors may be due to genetic factors such as mutations, deletions, and insertions (Fog et al. 2015), as well as epigenetic modifications (Casamassimi et al., 2020; Di Donato et al. 2023). PRDM5, for instance, has been identified as a transcriptional repressor through its interaction with histone methyltransferase G9a (Duan et al. 2007; Mehmood et al. 2020). PRDM14 may play a crucial role in maintaining cellular pluripotency, with its aberrant expression potentially linked to the progression of breast cancer (Dettman and Justice 2008). The EVI1 protein, encoded by the MECOM gene, is a multifunctional transcription factor critical in various hematological malignancies, particularly acute myeloid leukemia (AML). Aberrant expression of EVI1 is associated with poor prognosis, as AML patients with high EVI1 expression typically exhibit a poor response to treatment and shorter survival (Paredes et al. 2022).

Due to their critical roles in cancer, MECOM and PRDM family members are considered potential therapeutic targets. Research directions include developing direct inhibitors of these gene-encoded proteins and exploring their upstream or downstream signaling pathways as intervention points. However, translating these findings into clinical applications requires a deeper understanding of these proteins' functions and the ability to develop specific targeted therapies. Considering the close association between the PRDM gene family and various malignancies, we hypothesize that the PRDM gene family may also be involved in UCEC development and could serve as an effective prognostic biomarker. It could help identify new therapeutic targets for UCEC, ultimately reducing its mortality rate.

The relationship between the TGF-beta signaling pathway and cancer is a significant research focus. Zhang et al. (2021); Jiang and Deng (2019) TGF-beta (Transforming Growth Factor-beta) signaling plays a complex and multifaceted role in the development of cancer (Zhang et al. 2021; Jiang and Deng 2019). On the one hand, TGF-beta typically acts as a tumor suppressor in normal cells, regulating cell proliferation, differentiation, and apoptosis to maintain a normal cellular state (Ben-Aharon et al. 2019; Bévant et al. 2022). However, in cancer cells, the activation of the TGF-beta signaling pathway is often associated with the progression and worsening of tumors, promoting cancer cells' proliferation, invasion, and metastasis (Liu et al. 2022; Zhang et al. 2022).

This study aims to investigate the PRDM gene family, particularly the role of MECOM (MDS1 and EVI1 Complex Locus Protein), in uterine corpus endometrial carcinoma and its underlying molecular mechanisms. Special attention will be given to the expression pattern of MECOM, its methylation status, and its relationship with clinicopathological characteristics of uterine corpus endometrial carcinoma. Our research will employ comprehensive bioinformatics analysis, utilizing the latest public resources for comprehensive evaluation. This analysis will include transcriptome analysis, survival analysis, gene set enrichment analysis (GSEA), protein–protein interaction (PPI), and co-expression analysis. By identifying reliable biomarkers for predicting the prognosis of uterine corpus endometrial carcinoma, we aim to facilitate personalized clinical management. Through our research findings, we hope to provide new insights into the molecular pathological mechanisms underlying uterine corpus endometrial carcinoma, establish a theoretical foundation for developing novel therapeutic strategies targeting this disease, and contribute to improving patient survival rates and quality of life.

## Materials and methods

### Data collection

We obtained 548 uterine corpus endometrial carcinoma (UCEC) samples from the TCGA database and downloaded the clinical data of UCEC patients from the TCGA GDC data portal (https://portal.gdc.cancer.gov/). Types of endometrial cancer include adenomas and adenocarcinomas, cystic, mucinous and serous neoplasms, and epithelial neoplasms, NOS (not otherwise specified).

#### UALCAN database

Based on the TCGA, MET500, and CPTAC data, we utilized the UALCAN resource (Chandrashekar et al. 2017), a cancer OMICS data integration analysis network, to comprehensively analyze the PRDM genes in UCEC and standard samples. This analysis assessed mRNA expression levels, protein expression status, and promoter methylation status. In addition, we explored the correlation between these indicators and clinical pathological parameters. Using this research design, we obtained comprehensive data support and a deeper understanding of the molecular characteristics of UCEC and its relationship with disease progression.

#### Analysis of human protein atlas (HPA) data

The Human Protein Atlas (https://www.proteinatlas.org/) is a valuable resource for studying the expression of human proteins in different tissues and cell lines, including normal endometrium and 50 UCEC tissue. In this study, we accessed the immunohistochemistry images of the PRDM protein in these tissues using the information provided by this database (Asplund et al. 2012). Cellular expression intensity was categorized based on staining color depth into different levels: no staining (0), weak (1 +), moderate (2 +), and intense (3 +). The percentage of cells at each intensity level was estimated by observing tissue sections under a microscope, estimating the percentage at each intensity level relative to the total number of cells. The H-score was calculated by multiplying the percentage of cells at each intensity level by the corresponding intensity level and then summing the results. The mathematical expression is as follows: H − score = ∑i = 1n (pi × si)H − score = i = 1∑n (pi × si), where pipi is the percentage of cells at intensity level iii (in percent form), sisi is the intensity value for level ii (typically 1, 2, 3, etc.), and nn is the number of intensity levels. The H-score evaluated the Expression using the following categorization: H-Score 0 to < 100 as low; H-Score 100–200 as intermediate; H-Score > 200–300 as high.

We analyzed these images and obtained quantitative data and insights into PRDM protein expression localization, particularly in UCEC. This information is crucial for further investigations into the function and relevance of PRDM protein about UCEC.

#### GEPIA data analysis

GEPIA (http://gepia.cancer-pku.cn/index.html) is a web-based tool that leverages data from the TCGA and GTEx projects, encompassing 9736 tumor samples and 8587 standard samples (Tang et al. 2017). In our study, we employed the "single gene analysis" module within GEPIA to assess the mRNA expression of the PRDM gene across various tissues. This particular analysis provides valuable insights into the discrepancies in PRDM gene expression between tumor and normal tissues, thereby shedding light on its potential role in cancer development.

#### LinkedOmics

LinkedOmics (http://www.linkedomics.org) is a unique portal website that offers comprehensive multi-omics data analysis from 32 TCGA cancer types and 10 Clinical Proteomic Tumor Analysis Consortium (CPTAC) cancer cohorts. In our study, we employed the “LinkInterpreter” module to investigate the involvement of PRDM family members in KEGG pathway enrichment analysis. Gene set enrichment analysis (GSEA) allowed us to analyze the enrichment of genes within predefined gene sets using the TCGA_UCEC dataset. The GSEA was performed with a minimum gene set size of 3 and 500 simulations. To evaluate the statistical significance of the results, we utilized the Spearman correlation test and applied a significance *p*-value truncation of 0.05 to identify the relevant pathway enrichments associated with PRDM family members. This analysis approach gives us insight into the importance of PRDM family members in specific pathway enrichments, shedding light on their potential roles in cancer development and progression.

#### CBioPortal data analysis

CBioPortal (https://www.cbioportal.org/) is a freely accessible web resource that provides extensive multidimensional cancer genomics data. It includes data from 5000 tumor samples sourced from 20 different cancer studies. For our study, we focused on analyzing 529 patients with uterine corpus endometrial carcinoma (UCEC) from The Cancer Genome Atlas (TCGA) and PanCancer Atlas datasets.

To assess the mRNA expression levels, we applied a z-score threshold of ± 2.0 to determine the significance of gene expression changes. The mRNA expression z-scores were derived from RNA Seq V2 RSEM data. Moreover, we employed the OncoPrint sub-tool within cBioPortal to visually represent each patient's genetic alteration profiles of PRDM genes.

In addition to the above analysis, we performed a Comparison/Survival analysis to investigate the relationship between genetic variations in the PRDM gene family and UCEC histological grade, subtypes, and tumor types. The objective was to comprehensively understand the genetic variations within the PRDM gene family in UCEC and their potential clinical implications.

Through these analyses, we gained valuable insights into the genetic variations in the PRDM gene family in UCEC and their connections to clinical features. These findings contribute to a better understanding of the underlying molecular mechanisms in UCEC and potentially guide future research and treatment strategies.

#### GeneMANIA data analysis

GeneMANIA (http://genemania.org/) is a powerful prediction server that serves as an integration platform for genomics and proteomics data obtained from various public biological datasets. This valuable tool offers comprehensive information on various biological interactions, including protein–protein, protein-DNA, and gene interactions. It also provides insights into pathways, gene and protein expression data, co-localization, and protein domains. In the context of our study, we leveraged this extensive database to construct a protein–protein interaction (PPI) network encompassing the PRDM family. By examining this network, we aim to gain a deeper understanding of the intricate relationships between different members of the PRDM family and explore their potential roles in cellular function and disease mechanisms.

#### TIMER database analysis

TIMER (https://cistrome.shinyapps.io/timer/), an online server, offers a comprehensive resource for immune infiltration analysis of 10,897 tumors from 32 cancer types (Li et al. 2017). In our study on uterine corpus endometrial carcinoma (UCEC), we utilized TIMER's “Gene” submodule to assess the association between PRDM gene expression levels and immune cell infiltration. This investigation focused on various immune cell types, including B cells, CD8 T cells, CD4 T cells, macrophages, neutrophils, and dendritic cells.

### Cell line selection and culture conditions

To evaluate the expression of MECOM in cancer cells, we investigated six cell lines obtained from the American Type Culture Collection (ATCC, USA, https://www.atcc.org/): HEC-1-A (Catalog Number: HTB-112), AN3 CA (Catalog Number: HTB-111), HEC-1B (Catalog Number: HTB-113), RL95-2 (Catalog Number: RL95-2), KLE (Catalog Number: CRL-1622), and a normal cervical epithelial cell line VK2/E6E7 (Catalog Number: CRL-2616).

The cells were cultured under specific conditions as follows: HEC-1-A cells were cultured in McCoy's 5A medium (Catalog Number: 30-2007, ATCC, USA) supplemented with 10% fetal bovine serum (Catalog Number: 16140089, Gibco, USA) and 1% penicillin–streptomycin (Catalog Number: 30-2300, ATCC, USA). AN3 CA and HEC-1B cells were cultured in Eagle's Minimum Essential Medium (EMEM) (Catalog Number: 30-2003, ATCC, USA) supplemented with 10% fetal bovine serum (Catalog Number: 16140089, Gibco, USA) and 1% penicillin–streptomycin (Catalog Number: 30-2300, ATCC, USA). RL95-2, KLE and VK2/E6E7 cells were cultured in DMEM: F-12 Medium (Catalog Number: 30-2006, ATCC, USA) supplemented with 10% fetal bovine serum (Catalog Number: 16140089, Gibco, USA) and 1% penicillin–streptomycin (Catalog Number: 30-2300, ATCC, USA). All cell lines were incubated in a humidified incubator at 37 °C with 5% CO_2_.

To examine the expression of MECOM, we employed RT-PCR (Table 1) and western blot techniques to detect the target proteins, MECOM (Catalog Number: MA5-11,144, Thermo Fisher Scientific, USA) and GAPDH (Catalog Number: 2118S, Cell Signaling Technology, USA). The primary antibodies were incubated overnight at 4 °C, followed by washing the membrane with 1 × TBST buffer four times for 8 min each. Subsequently, rabbit secondary antibodies (Catalog Number: 7074P2, Cell Signaling Technology, USA) were incubated at room temperature for two hours, and the membrane was washed four more times with 1 × TBST buffer for 8 min each.

**Table 1:** The primer sequence of qRT-RCR

Target	Sequence (5'-3')
MECOM	F: AATATGAGTCATGCCAACCC
	R: CTTGGTGTACTGACATCATC
GAPDH	F: TGCACCACCAACTGCTTA
	R: GGATGCAGGGATGATGTTC
PRDM1	F: AAGCAACTGGATGCGCTATGT
	R: GGGATGGGCTTAATGGTGTAGAA
PRDM2	F: AAGTGAGCCGAGTTTCACCTC
	R: CTAATCGCTCGTCTGGTTCTG
PRDM4	F: TCCTCTGTGAGCAATGCCTTG
	R: CCACACATCACCCCTCGAT
PRDM5	F: TACGTGCCGGACAGGTTCT
	R: TTCACCCTTTCGCACTCTGC
PRDM6	F: GGTGGGGAACCTAGTAAGTCG
	R: ACCGTTGAAGGGACATTTAAGTT
PRDM7	F: GCAGGGAGAGAACCAAAGCC
	R: ACTTATCACTGAAACCTTGCCC
PRDM8	F: TTTTACCAGCGTTTACACCACC
	R: GCTGTCATATAGGGAAGTATGGC
PRDM9	F: CAGCCAACAATGGATACTCCTG
	R: CTGGCCGTATTCATCCCCA
PRDM10	F: GCTGCCTTCCATCGAGAGTG
	R: CCAGTCATCCAGATCCGTGTC
PRDM11	F: GTCTGCTCACCACTCCGAG
	R: TTGGGCATTCATCCACGAAGT
PRDM12	F: CAAGGCGGGAACCGAGATG
	R: CACATGAGGTTGTTGTTCTTGC
PRDM13	F: TGGAGTGGATAGGGTTAATCCG
	R: CGGGTAAGTCTGCAATAGCTTC
PRDM14	F: ACACGCCTTTCCCGTCCTA
	R: GGGCAGATCGTAGAGAGGCT
PRDM15	F: ACTTGGAGATCAGACGACTGG
	R: TGGACTCAAAGGGACCGAACT
PRDM16	F: CGAGGCCCCTGTCTACATTC
	R: GCTCCCATCCGAAGTCTGTC

Finally, the intensity of the protein bands was measured using an ECL reagent (Catalog Number: 34580, Thermo Fisher Scientific, USA) and the Bio-Rad gel imaging system. The resulting images were analyzed using ImageJ's image analysis software to determine the interaction between MECOM and the target proteins. Statistical analysis was performed using *t*-tests to compare differences between different groups, and each experiment was conducted in triplicate.

### CRISPR-Cas9 gene knockout

The MECOM knockout cells in HEC-1-A cells were generated using the CRISPR/Cas9. The Lenti-CRISPR v2 vector (Hanbio, Shanghai, China) containing the Streptococcus pyogenes Cas9 nuclease gene was utilized to deliver the sgRNA. The sgRNA sequence used for knockout is provided in Table 2. HEC-1-A cells were transduced with the lentiviral vector and subsequently subjected to CRISPR/Cas9 editing to create MECOM knockout cells. To select transfected cells with sgRNA plasmids and donor sequences, 4 μg/mL puromycin (Catalog Number: 540222, Sigma-Aldrich, USA) was used. The specific sequences for the sgRNA and donor are given in Table 2. Surviving cells from limited dilution cloning were chosen, and the knockout of MECOM in HEC-1-A cells was confirmed through RT-qPCR analysis.

**Table 2:** The CRISPR guide RNA sequence

Name	Sequence (5 '-3')
V2-MECOM-1	CACCGGCGTGAGTGGTACTAACCGTGG
V2-MECOM-2	AAACCCACGGTTAGTACCACTCACGCC

### Construction of lentiviral overexpression vectors and experimental grouping

The pLVX-Puro vector (pLVX-, overexpression vector, Catalog Number: 632164, Clontech, USA) created a lentiviral-based MECOM overexpression construct and a negative control. Lentiviral particles carrying the vector were generated in HEK-293 T cells using the lentiviral packaging kit (Catalog Number: A35684CN, Invitrogen, USA). The iCell-h237 HEK-293 T cell line (Catalog Number: iCell-h237, Cyagen Biosciences, Shanghai, China) was used for the experiments. The lentivirus supernatant was collected 48 h after transfection and had a 1 × 10^8^ TU/mL titer.

To infect the cells with the lentivirus, 1 × 10^5^ cells were seeded in a 6-well plate. When the cells reached 60–70% confluence, the culture medium was supplemented with the appropriate amount of packaged lentivirus (MOI = 1, working titer approximately 1 × 10^5^ TU/mL) and 5 μg/mL polybrene (Catalog Number: TR-1003, Merck, USA). After 4 h of transfection, the polybrene was diluted by adding an equal volume of culture medium. After 24 h of transfection, the fresh culture medium was replaced. Puromycin (Catalog Number: A1113803, Thermo Fisher) at a concentration of 1 μg/mL was used for selection to obtain stable transfected cell lines. The specific overexpression sequence details can be found in Table 3.

**Table 3:** Overexpression sequences

MECOM-CDS sequence (5'-3') NM_001105078.4	
ATGAAGAGCGAAGACTATCCCCATGAAACTATGGCGCCGGATATCCACGAAGAACGGCAATATCGCTGCGAAGACTGTGACCAGCTCTTTGAATCTAAGGCTGAACTAGCAGATCACCAAAAGTTTCCATGCAGTACTCCTCACTCAGCATTTTCAATGGTTGAAGAGGACTTTCAGCAAAAACTCGAAAGCGAGAATGATCTCCAAGAGATACACACGATCCAGGAGTGTAAGGAATGTGACCAAGTTTTTCCTGATTTGCAAAGCCTGGAGAAACACATGCTGTCACATACTGAAGAGAGGGAATACAAGTGTGATCAGTGTCCCAAGGCATTTAACTGGAAGTCCAATTTAATTCGCCACCAGATGTCACATGACAGTGGAAAGCACTATGAATGTGAAAACTGTGCCAAGGTTTTCACGGACCCTAGCAACCTTCAGCGGCACATTCGCTCTCAGCATGTCGGTGCCCGGGCCCATGCATGCCCGGAGTGTGGCAAAACGTTTGCCACTTCGTCGGGCCTCAAACAACACAAGCACATCCACAGCAGTGTGAAGCCCTTTATCTGTGAGGTCTGCCATAAATCCTATACTCAGTTTTCAAACCTTTGCCGTCATAAGCGCATGCATGCTGATTGCAGAACCCAAATCAAGTGCAAAGACTGTGGACAAATGTTCAGCACTACGTCTTCCTTAAATAAACACAGGAGGTTTTGTGAGGGCAAGAACCATTTTGCGGCAGGTGGATTTTTTGGCCAAGGCATTTCACTTCCTGGAACCCCAGCTATGGATAAAACGTCCATGGTTAATATGAGTCATGCCAACCCGGGCCTTGCTGACTATTTTGGCGCCAATAGGCATCCTGCTGGTCTTACCTTTCCAACAGCTCCTGGATTTTCTTTTAGCTTCCCTGGTCTGTTTCCTTCCGGCTTGTACCACAGGCCTCCTTTGATACCTGCTAGTTCTCCTGTTAAAGGACTATCAAGTACTGAACAGACAAACAAAAGTCAAAGTCCCCTCATGACACATCCTCAGATACTGCCAGCTACACAGGATATTTTGAAGGCACTATCTAAACACCCATCTGTAGGGGACAATAAGCCAGTGGAGCTCCAGCCCGAGAGGTCCTCTGAAGAGAGGCCCTTTGAGAAAATCAGTGACCAGTCAGAGAGTAGTGACCTTGATGATGTCAGTACACCAAGTGGCAGTGACCTGGAAACAACCTCGGGCTCTGATCTGGAAAGTGACATTGAAAGTGATAAAGAGAAATTTAAAGAAAATGGTAAAATGTTCAAAGACAAAGTAAGCCCTCTTCAGAATCTGGCTTCAATAAATAATAAGAAAGAATACAGCAATCATTCCATTTTCTCACCATCTTTAGAGGAGCAGACTGCGGTGTCAGGAGCTGTGAATGATTCTATAAAGGCTATTGCTTCTATTGCTGAAAAATACTTTGGTTCAACAGGACTGGTGGGGCTGCAAGACAAAAAAGTTGGAGCTTTACCTTACCCTTCCATGTTTCCCCTCCCATTTTTTCCAGCATTCTCTCAATCAATGTACCCATTTCCTGATAGAGACTTGAGATCGTTACCTTTGAAAATGGAACCCCAATCACCAGGTGAAGTAAAGAAACTGCAGAAGGGCAGCTCTGAGTCCCCCTTTGATCTCACCACTAAGCGAAAGGATGAGAAGCCCTTGACTCCAGTCCCCTCCAAGCCTCCAGTGACACCTGCCACAAGCCAAGACCAGCCCCTGGATCTAAGTATGGGCAGTAGGAGTAGAGCCAGTGGGACAAAGCTGACTGAGCCTCGAAAAAACCACGTGTTTGGGGGAAAAAAAGGAAGCAACGTCGAATCAAGACCTGCTTCAGATGGTTCCTTGCAGCATGCAAGACCCACTCCTTTCTTTATGGACCCTATTTACAGAGTAGAGAAAAGAAAACTAACTGACCCACTTGAAGCTTTAAAAGAGAAATACTTGAGGCCTTCTCCAGGATTCTTGTTTCACCCACAATTCCAACTGCCTGATCAGAGAACTTGGATGTCAGCTATTGAAAACATGGCAGAAAAGCTAGAGAGCTTCAGTGCCCTGAAACCTGAGGCCAGTGAGCTCTTACAGTCAGTGCCCTCTATGTTCAACTTCAGGGCGCCTCCCAATGCCCTGCCAGAGAACCTTCTGCGGAAGGGAAAGGAGCGCTATACCTGCAGATACTGTGGCAAGATTTTTCCAAGGTCTGCAAACCTAACACGGCACTTGAGAACCCACACAGGAGAGCAGCCTTACAGATGCAAATACTGTGACAGATCATTTAGCATATCTTCTAACTTGCAAAGGCATGTTCGCAACATCCACAATAAAGAGAAGCCATTTAAGTGTCACTTATGTGATAGGTGTTTTGGTCAACAAACCAATTTAGACAGACACCTAAAGAAACATGAGAATGGGAACATGTCCGGTACAGCAACATCGTCGCCTCATTCTGAACTGGAAAGTACAGGTGCGATTCTGGATGACAAAGAAGATGCTTACTTCACAGAAATTCGAAATTTCATTGGGAACAGCAACCATGGCAGCCAATCTCCCAGGAATGTGGAGGAGAGAATGAATGGCAGTCATTTTAAAGATGAAAAGGCTTTGGTGACCAGTCAAAATTCAGACTTGCTGGATGATGAAGAAGTTGAAGATGAGGTGTTGTTAGATGAGGAGGATGAAGACAATGATATTACTGGAAAAACAGGAAAGGAACCAGTGACAAGTAATTTACATGAAGGAAACCCTGAGGATGACTATGAAGAAACCAGTGCCCTGGAGATGAGTTGCAAGACATCCCCAGTGAGGTATAAAGAGGAAGAATATAAAAGTGGACTTTCTGCTCTAGATCATATAAGGCACTTCACAGATAGCCTCAAAATGAGGAAAATGGAAGATAATCAATATTCTGAAGCTGAGCTGTCTTCTTTTAGTACTTCCCATGTGCCAGAGGAACTTAAGCAGCCGTTACACAGAAAGTCCAAATCGCAGGCATATGCTATGATGCTGTCACTGTCTGACAAGGAGTCCCTCCATTCTACATCCCACAGTTCTTCCAACGTGTGGCACAGTATGGCCAGGGCTGCGGCGGAATCCAGTGCTATCCAGTCCATAAGCCACGTATGA	

The overexpression of MECOM was confirmed using RT-qPCR. The HEC-1-A cells were divided into the following experimental groups based on the experimental requirements: pLVX-NC (overexpression control group), pLVX-MECOM (MECOM overexpression group), V2-NC (knockout control group), V2-MECOM (MECOM knockout group).

#### RT-qPCR

Total RNA was extracted from the tissues using Trizol (Invitrogen, USA) according to the manufacturer's instructions (Catalog Number: 16096020). Reverse transcription was performed using the reverse transcription kit from Takara (Japan) to synthesize cDNA for mRNA detection (Catalog Number: RR047A). Quantitative real-time PCR (qRT-PCR) was conducted using the TaqMan Gene Expression Assays protocol (Applied Biosystems, USA). GAPDH was used as the internal control. The qRT-PCR cycling program consisted of an initial denaturation step at 95 °C for 10 min, followed by 35 cycles of denaturation at 95 °C for 15 s, annealing at 60 °C for 30 s, and extension at 72 °C for 45 s. Three replicates were performed for each qRT-PCR. The primer information can be found in Table 1. The relative expression level of the target gene in the experimental group compared to the control group was calculated using the 2^−ΔΔCt^ method. The formula used was ΔΔCT = ΔCt experimental group -ΔCt control group, where ΔCt = Ct target gene -Ct internal control gene. Ct represents the cycle number at which the real-time fluorescence intensity reaches the set threshold during amplification, indicating the exponential phase of amplification. The experiments were repeated three times.

### Detection of cell proliferation capacity using CCK-8 assay

The CCK-8 assay kit (Catalog Number: WH1199, Shanghai Wei'ao Biotechnology Co., Ltd., Shanghai, China) was utilized to measure cell proliferation. Cells in the logarithmic growth phase were adjusted to a 5 × 10^4^ cells/mL concentration in McCoy's 5A medium supplemented with 10% FBS. Subsequently, the cells were seeded in a 96-well culture plate, adding 100 μL of cell culture medium into each well, and incubated in a cell culture incubator for 24 h, 48 h, and 72 h, respectively.

Following removal of the culture medium, 10 μL of CCK-8 solution was added to each well, then incubated at 37 °C for 2 h. The absorbance value (A) was measured at a wavelength of 450 nm using a Multiskan FC Microplate Reader (Catalog Number: 51119080, Thermo Fisher Scientific, USA). The proliferation rate (%) was calculated according to the formula [(A control group—A experiment group)/A control group] × 100%. Each group was analyzed in triplicate, and the average value was calculated. Each experiment was repeated three times.

### Detection of cell apoptosis using flow cytometry

Apoptosis of cells in the HEC-1-A cell line was assessed using the Annexin V-FITC/PI double staining method. Following different treatments, the cells were collected in a 15 mL centrifuge tube and centrifuged at 800 g. The resulting supernatant was then discarded. The cell pellets were washed twice with PBS and resuspended in 500 μL of binding buffer, per the instructions in the cell apoptosis detection kit (Catalog Number: 556547, BD Bioscience, USA).

The staining process involved adding 5 μL each of FITC and PI to the cells. The cells were mixed thoroughly and incubated in the dark for 15 min. Following incubation, the cells were analyzed for apoptosis using a BD FACSCalibur flow cytometer. Cells that tested positive for Annexin V-FITC were considered apoptotic. This experiment was repeated three times to ensure the reliability of the results.

### Detection of cell cycle using flow cytometry

Obtaining HEC-1-A cells treated through various methods involved first fixing the cells in pre-chilled 75% ethanol for at least one hour. Subsequently, a single wash with PBS solution was performed. For staining, 400 μl of PI staining solution (50 μg/ml, Catalog Number: P4170, Sigma-Aldrich, USA) and 100 μl of RNase A (100 μg/ml, RNASEA-RO, Sigma-Aldrich, USA) were added. The staining was conducted in the dark at 4 °C for 30 min, followed by flow cytometer analysis using a BD Biosciences instrument (USA). Cell cycle fitting software ModFit was employed for data analysis. Each experiment was conducted in triplicate.

### Detection of cell migration using transwell assay

In the migration experiment, HEC-1-A cell suspension with different treatments (200 μL) was added to the upper chamber of each well, while 800 μL of conditioned medium containing 20% FBS was added to the lower chamber. The plates were then incubated at 37 °C for 24 h. After incubation, the Transwell chambers were removed, and the inner layer of the Transwell membrane was gently wiped with a cotton swab. The membrane was then washed twice with PBS. Next, the cells in the chambers were fixed with 4% formaldehyde and washed thrice with water. Afterward, the cells were incubated with 0.1% crystal violet for 30 min. Images were captured to assess migration using a Nikon Eclipse Ci optical microscope (Nikon, Tokyo, Japan). Cell counting was performed in five different areas of each sample. This process was repeated three times for each sample to ensure accuracy in cell quantification.

### Western blot

To perform protein electrophoresis separation, Bio-Rad's Mini-PROTEAN TGX gels (Catalog No.: 4561096, Bio-Rad, USA) were used, diluted into 1 × Tris-based buffer (20 × Tris–Glycine buffer, Catalog No.: LC2675, Invitrogen, USA) and electrophoresed using the Bio-Rad Mini-PROTEAN Tetra system. Subsequently, the separated proteins were transferred onto PVDF membranes (Catalog No.: ISEQ00010, Sigma-Aldrich, GER) using the Trans-Blot Turbo Transfer Buffer (Catalog No.: 1704272, Bio-Rad, USA). Following this, the membranes were blocked at room temperature with 5% non-fat milk powder (Catalog No. 1706404, Bio-Rad, USA) for one hour. Then, the membranes were washed four times with 1 × TBST buffer (Catalog No.: 9997S, Cell Signaling Technology, USA), each wash lasting eight minutes.

Next, we explored the specific molecular mechanisms of apoptosis and cell cycle by conducting Western blot analysis. Specific antibodies were used to detect target proteins, including MECOM (Catalog Number: 2593, Cell Signaling Technology, USA), Smad2/3 (Catalog No.: 8685S, Cell Signaling Technology, USA), BAX (Catalog No.: 5023S, Cell Signaling Technology, USA), CASP3 (Catalog No.: 14220S, Cell Signaling Technology, USA), BCL2 (Catalog No.: 4223S, Cell Signaling Technology, USA), and BCL-XL (Catalog No.: 2764S, Cell Signaling Technology, USA), CHK1 (Catalog No.: 37010S, Cell Signaling Technology, USA), CHK2 (Catalog No.: 6334S, Cell Signaling Technology, USA), CDK2 (Catalog No.: 18048S, Cell Signaling Technology, USA), and P21 (Catalog No.: 2947S, Cell Signaling Technology, USA). These antibodies were incubated overnight at 4 °C, followed by washing the membranes four times with 1 × TBST buffer, each wash lasting eight minutes. The secondary antibody from the rabbit (Catalog No.: 7074P2, Cell Signaling Technology, USA) was incubated at room temperature for two hours, followed by another four washes with 1 × TBST buffer, each lasting eight minutes.

Finally, the intensity of protein bands was quantified using ECL detection liquid (Catalog No.: 34580, Thermo Fisher Scientific, USA) and the Bio-Rad gel imaging system, along with ImageJ software for image analysis, to confirm the interaction of MECOM with the target proteins.

### Protein immunoprecipitation (co-IP)

The following steps were taken to perform protein immunoprecipitation experiments using the Co-IP kit (Catalog Number: 88804, Thermo Fisher Scientific, USA). First, the HEC-1-A cell line was cultured, including pLVX-NC (overexpression control group) and pLVX-MECOM (overexpression MECOM group) cells, until near confluence. The cells were then lysed using RIPA buffer containing proteinase inhibitors to obtain the cell lysate. The protein concentration in the lysate was measured using the Pierce BCA Protein Assay Kit (Catalog Number: 23227, Thermo Fisher Scientific, USA) to determine the protein content of the samples. A/G beads were used to remove non-specific protein binding by treating the lysate. To form the antibody-protein complex, the lysate was mixed with an antibody against MECOM (Catalog Number: 2593, Cell Signaling Technology, USA) and incubated overnight at 4 °C. Protein A/G beads were then added and incubated for several hours to facilitate binding the antibody-protein complex to the beads. The beads were subsequently washed with PBS or RIPA buffer to remove non-specifically bound proteins.

For protein gel separation, Bio-Rad's Mini-PROTEAN TGX gel (Catalog Number: 4561096, Bio-Rad, USA) was used, diluted in 1 × Tris-based buffer (20 × Tris–Glycine buffer, Catalog Number: LC2675, Invitrogen, USA), and the Bio-Rad Mini-PROTEAN Tetra system was used for electrophoresis. The separated proteins were transferred onto a PVDF membrane (ISEQ00010, Sigma-Aldrich, GER) using a Trans-Blot Turbo Transfer Buffer (Catalog Number: 1704272, Bio-Rad, USA). The membrane was then blocked with 5% skim milk (Catalog Number: 1706404, Bio-Rad, USA) at room temperature for one hour. Subsequently, the membrane was washed four times with 1 × TBST buffer (Catalog Number: 9997S, Cell Signaling Technology, USA) for 8 min each.

For western blot analysis, specific antibodies were used to detect target proteins, including MST1 (Catalog Number: 3682S, Cell Signaling Technology, USA), LATS1 (Catalog Number: 3477S, Cell Signaling Technology, USA), YAP (Catalog Number: 14074S, Cell Signaling Technology, USA), SAV1 (Catalog Number: 13301S, Cell Signaling Technology, USA), and MOB1 (Catalog Number: 13730S, Cell Signaling Technology, USA). These antibodies were incubated overnight at 4 °C, followed by washing the membrane four times with 1 × TBST buffer for 8 min each. A secondary antibody (Catalog Number: 7074P2, Cell Signaling Technology, USA), raised in rabbits, was then incubated with the membrane at room temperature for two hours, followed by another round of washing the membrane four times with 1 × TBST buffer for 8 min each.

To confirm the interaction between MECOM and target proteins, we utilized ECL fluorescent liquid (Catalog Number: 34580, Thermo Fisher Scientific, USA), along with a Bio-Rad gel imaging system and ImageJ image analysis software. The intensity of protein bands was quantified using these tools. Statistical analysis was performed using t-tests, with each experiment repeated three times.

Moving on, we delved into the specific molecular mechanisms of apoptosis and the cell cycle by employing Western blot analysis. Target proteins, including MST1 (Catalog Number: 3682S, Cell Signaling Technology, USA), BAX (Catalog Number: 5023S, Cell Signaling Technology, USA), CASP3 (Catalog Number: 14220S, Cell Signaling Technology, USA), BCL2 (Catalog Number: 4223S, Cell Signaling Technology, USA), and BCL-XL (Catalog Number: 2764S, Cell Signaling Technology, USA), were detected using specific antibodies. CHK1 (Catalog Number: 37010S, Cell Signaling Technology, USA), CHK2 (Catalog Number: 6334S, Cell Signaling Technology, USA), CDK2 (Catalog Number: 18048S, Cell Signaling Technology, USA), and P21 (Catalog Number: 2947S, Cell Signaling Technology, USA) antibodies were also employed. These antibodies were incubated overnight at 4 °C, followed by four washes with 1 × TBST buffer for 8 min each.

Next, the membranes were exposed to rabbit secondary antibodies (Catalog Number: 7074P2, Cell Signaling Technology, USA) at room temperature for two hours. Then, they were washed four times with 1 × TBST buffer for eight minutes each.

To quantify the intensity of protein bands and confirm the MECOM-target protein interaction, we employed ECL fluorescent liquid (Catalog Number: 34580, Thermo Fisher Scientific, USA), the Bio-Rad gel imaging system, and ImageJ image analysis software. Statistical analysis was performed through *t*-tests to compare differences between different experimental groups. Each experiment was repeated three times.

#### Statistical analysis

For the analysis of bioinformatics, R software version 4.3.2 was primarily used for data processing and analysis. The differential expression of members of the PRDM family in UCEC (uterine corpus endometrial carcinoma) and normal tissues was determined using the Wilcoxon rank sum test. The relationship between PRDM expression and clinical pathological features was evaluated using the Kruskal–Wallis test. Logistic regression analysis was conducted to explore the correlation between PRDM expression and clinical parameters. Survival analysis involved using Cox proportional hazards regression models to assess the impact of various factors on overall survival. Additionally, Kaplan–Meier methods were employed to plot survival curves. ROC (receiver operating characteristic) analysis was performed to evaluate the potential diagnostic value of PRDMs in UCEC.

We primarily analyzed differences between experimental groups in the statistical analysis of cell experiments. *T*-tests or one-way analysis of variance (ANOVA) were used to compare the numbers of migrating cells among different experimental groups in cell migration experiments. Chi-square tests or Fisher's exact tests were applied to analyze the distribution differences of cells in each stage for cell cycle and apoptosis analysis. When experimental data involved multiple time points, repeated measures ANOVA or mixed-effects models were utilized to analyze the effects of time and its interaction with treatment groups. Ensuring the reliability of the results involved repeating all cell experiments at least three times.

Throughout the analysis process, a significance level of *p* < 0.05 was used to indicate statistical significance, while *p* < 0.01 was considered to have vital statistical significance.

## Results

### Abnormal expression analysis of PRDM family members in uterine *corpus* endometrial carcinoma

**Fig. 1:**
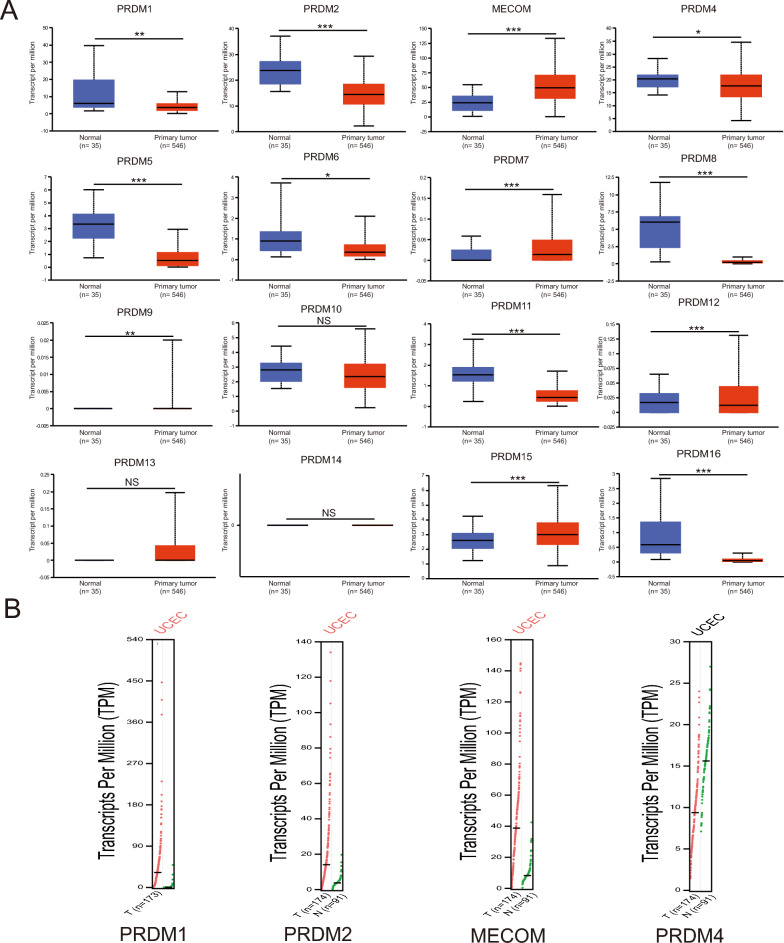

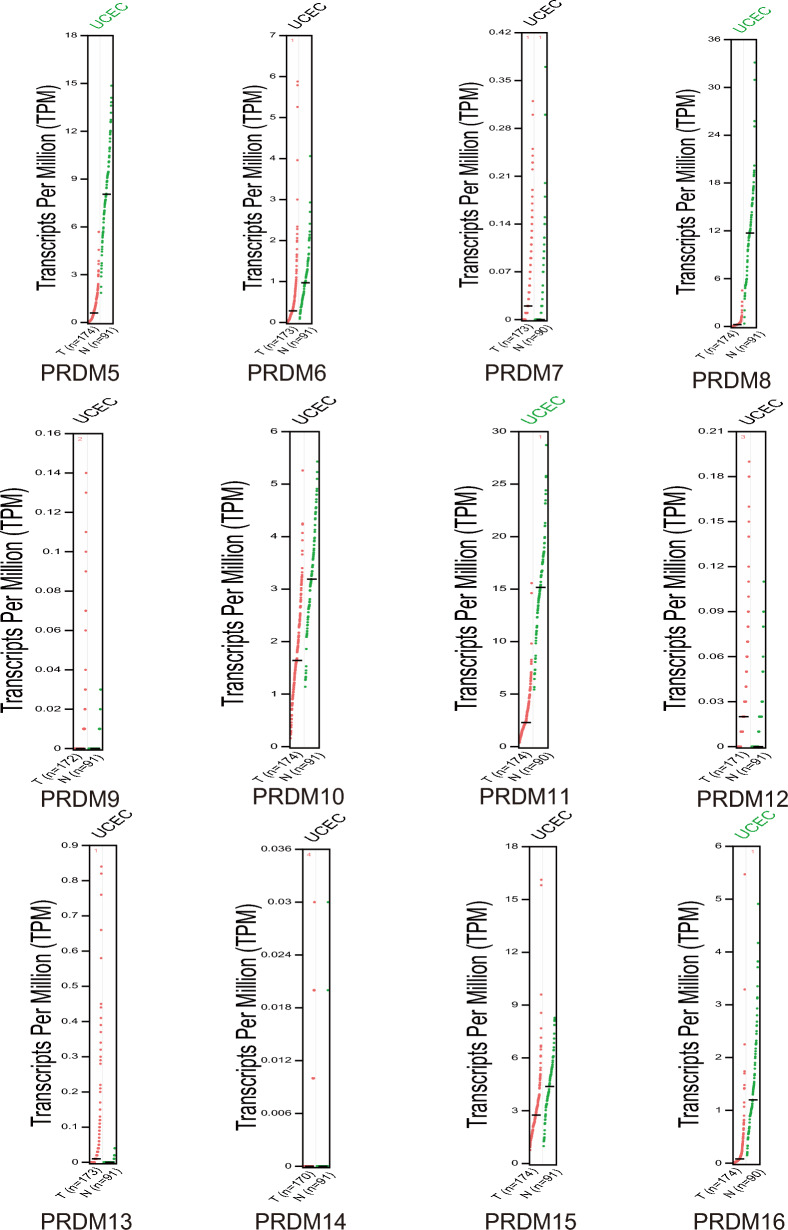
MRNA expression of PRDM family members in UCEC. **A** UALCAN analysis was performed to investigate the mRNA expression levels of PRDM family members in normal endometrial and tumor tissue. Our results revealed significant upregulation of MECOM, PRDM7, and PRDM15 in tumor tissue, while the expression of PRDM1, PRDM2, PRDM4, and PRDM5 was significantly downregulated. Statistical significance between different expression levels was indicated by asterisks (*p < 0.05, **p < 0.01, ***p < 0.001), and no significant difference was denoted as NS. **B** A GEPIA analysis was conducted to obtain a comprehensive overview of the expression profiles of the PRDM family members. Each dot in the figure represents an independent sample, with tumor tissue represented by red and normal tissue represented by green. This visual representation provides valuable insights into the overall expression patterns of the PRDM family

In this study, we used the UALCAN and GEPIA analysis tools to compare the transcript levels of PRDM family members in uterine corpus endometrial carcinoma (UCEC) and normal endometrial tissue. As depicted in Fig. 1A–B, our results reveal that MECOM, PRDM7, and PRDM15 transcription levels were significantly higher in UCEC tissue than in normal tissue. It indicates the potential role of these genes in tumor occurrence and development. Conversely, the mRNA expression of PRDM1, PRDM2, PRDM4, PRDM5, PRDM6, PRDM8, PRDM11, PRDM12, and PRDM16 in cancer tissue was notably lower than in normal tissue. The observed differences may be attributed to the regulatory nature of PRDM genes in processes such as cell proliferation, differentiation, and apoptosis, and their aberrant expression might be involved in the pathogenesis of uterine corpus endometrial carcinoma.

**Fig. 2:**
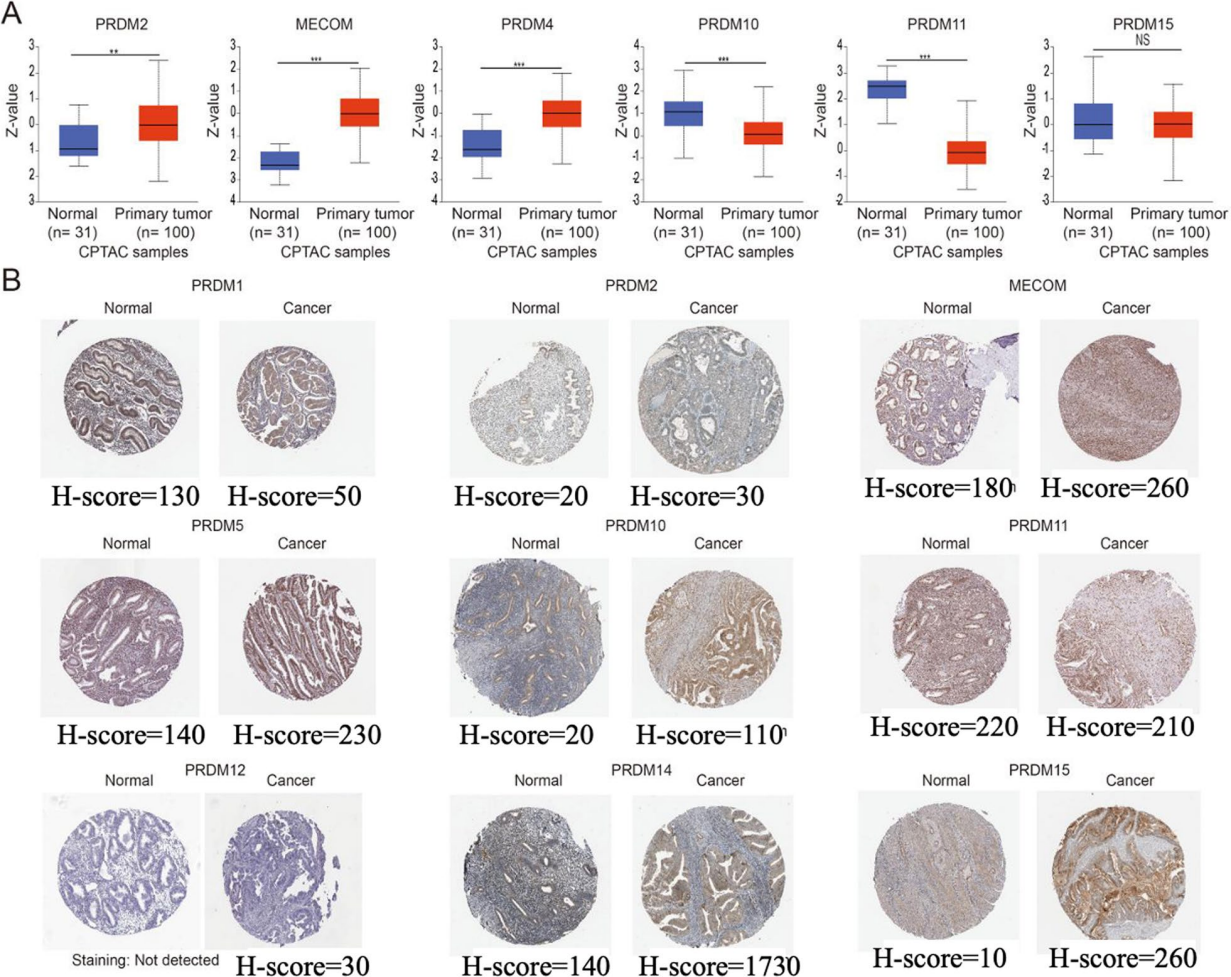
Protein expression levels of PRDM family members in uterine corpus endometrial carcinoma (UCEC) and normal endometrial tissue. **A** Protein levels of PRDM family members in uterine corpus endometrial carcinoma (UCEC) patients were analyzed using data from UALCAN. The results indicate significant differences in protein levels among the family members. Statistical significance was observed for *p* < 0.01 and *p < 0.001, while no significant difference (NS) was found in some cases. **B** MECOM, PRDM5, PRDM10, and PRDM11 expression patterns in UCEC and normal endometrial tissue were investigated using immunohistochemistry images in the HPA database. The images demonstrate these proteins' distinct localization and expression patterns in different tissues. PRDM5 exhibits a significantly higher H-score in cancer tissues compared to normal tissues. In contrast, PRDM12 is undetectable in normal tissues but presents a low H-score in cancer tissues

Furthermore, we investigated the protein levels of PRDMs in UCEC patients through the UALCAN database analysis (Fig. 2A). Intriguingly, we found that the protein expression levels of MECOM, PRDM5, PRDM10, and PRDM11 were generally higher in UCEC tissue compared to normal tissue, while the expression of PRDM1 was diminished in cancer tissue. It is worth noting that the protein expression data obtained from the HPA database needed to align entirely with the findings from UALCAN. As demonstrated in Fig. 2B, the H-score levels of the protein expression levels of MECOM and PRDM11 were significantly higher in UCEC tissue, whereas the H-scores of PRDM1 and PRDM12 were weakened or undetectable in cancer tissue. By utilizing immunohistochemistry images, we gained insights into the distribution and localization of these proteins in the tissue, which facilitated a better understanding of their distinct roles under normal and pathological conditions.

We noticed discrepancies between the data from UALCAN and HPA. UALCAN (http://ualcan.path.uab.edu/) primarily provides large-scale gene expression data for cancer patients and normal populations, including features like differential gene expression and survival analysis. The data come from TCGA and other public cancer genomics databases, focusing on mRNA expression levels. On the other hand, HPA (https://www.proteinatlas.org/) focuses on protein expression levels, utilizing antibody-mediated immunohistochemistry to visualize protein distribution in different human tissues. It also includes RNA expression data and single-cell expression data. Due to UALCAN and HPA focusing on mRNA and protein levels, they may show different expression profiles. mRNA expression levels do not always correlate with protein levels, as post-transcriptional modifications, translation efficiency, and protein degradation can affect the protein levels.

Collectively, our findings reinforce the notion of an overall increase in the mRNA and protein levels of MECOM in UCEC patients. It further highlights the significance of PRDM family members as potential regulatory factors in the occurrence and development of uterine corpus endometrial carcinoma, thus providing essential scientific evidence for future investigations into these genes' specific functions and mechanisms in uterine corpus endometrial carcinoma.

### Correlation between PRDM transcriptional expression and clinical pathological parameters and prognosis in UCEC patients

To evaluate the clinical significance of PRDMs' mRNA levels, we used UALCAN to examine the relationship between these levels and various clinical pathological parameters in UCEC patients. These parameters included the patients' cancer stage, weight, and menopausal status.

As demonstrated in Figure S1, the mRNA expression of PRDM family members showed a significant association with the patient's cancer stage. Generally, patients in later stages tended to exhibit lower expression of PRDMs mRNA. However, for PRDM14, no significant differences were observed except for MECOM, PRDM7, and PRDM15.

Additionally, in Figure S2, the mRNA expression of PRDMs in UCEC patients shows a specific correlation with normal endometrial tissue. Compared to normal endometrium, UCEC patients exhibit lower mRNA expression levels of MECOM, PRDM7, and PRDM15, and higher mRNA expression levels of PRDM1, PRDM2, PRDM5, PRDM6, PRDM8, PRDM11, and PRDM16. However, there is no significant difference in the mRNA expression of PRDMs between UCEC patients of different weights.

Furthermore, we investigated the expression of PRDMs in UCEC patients with different menopausal statuses. Figure S3 illustrated that the expression patterns of PRDMs, except PRDM13, were significantly correlated with menopausal status.

**Fig. 3:**
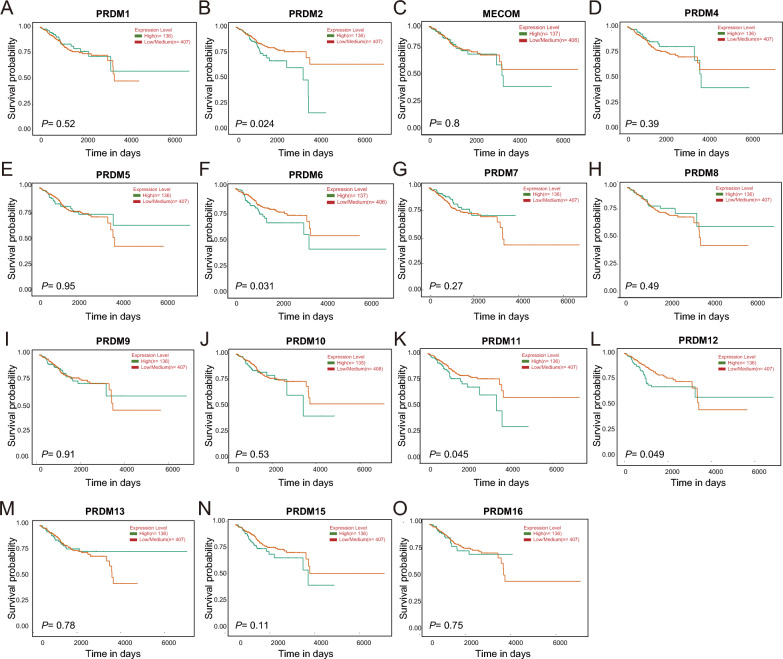
Survival analysis of the PRDM family members in UCEC (UALCAN). Generally, in patients with uterine corpus endometrial carcinoma (UCEC), higher expression of UCEC mRNA is associated with poorer overall survival (OS) (**A**, **C-E**, **G-J**, **L-O**). However, there is no significant prognostic correlation observed with the expression of PRDM1, MECOM, PRDM4, PRDM5, PRDM7, PRDM8, PRDM9, PRDM10, PRDM12, PRDM13, PRDM15, and PRDM16 (**A, C-E, G-J, L-O**). On the other hand, lower expression of PRDM2, PRDM6, and PRDM11 mRNA is significantly associated with improved OS, suggesting their potential as promising prognostic biomarkers (**B, F, K**)

Moving forward, we explored the predictive value of different PRDMs mRNA expressions in UCEC patients using the UALCAN server. As depicted in Fig. 3A–O, most PRDM family members were not significantly associated with patient prognosis. However, our findings indicate that lower PRDM2, PRDM6, and PRDM11 mRNA expression is associated with favorable survival rates in UCEC patients. Low PRDM2, PRDM6, and PRDM11 mRNA expression in UCEC patients is associated with better survival rates. However, compared to healthy individuals, PRDM2, PRDM6, and PRDM11 mRNA expression is downregulated in UCEC patients. At first glance, this seems paradoxical since PRDMs typically function as tumor suppressor genes in normal cells. However, once cancer has developed, the continuous low expression of PRDMs might no longer promote tumor progression. Instead, it may be associated with a more stable tumor state or less aggressive behavior. The low expression of PRDMs could be linked to enhanced immune surveillance, as PRDM2 expression might affect the immunogenicity of tumor cells. Tumors with low PRDM expression may be more easily recognized and eliminated by the immune system, thereby reducing tumor burden.

### Methylation status of PRDM family member promoters in UCEC patients

We conducted an analysis using the UALCAN server to investigate the potential role of promoter methylation in the downregulation of PRDM1, PRDM2, PRDM4, PRDM5, PRDM6, PRDM8, PRDM11, PRDM12, and PRDM16 mRNA expression in uterine corpus endometrial carcinoma (UCEC) tissues. Our analysis revealed exciting findings regarding the differential methylation patterns between normal and UCEC tissues.

**Fig. 4:**
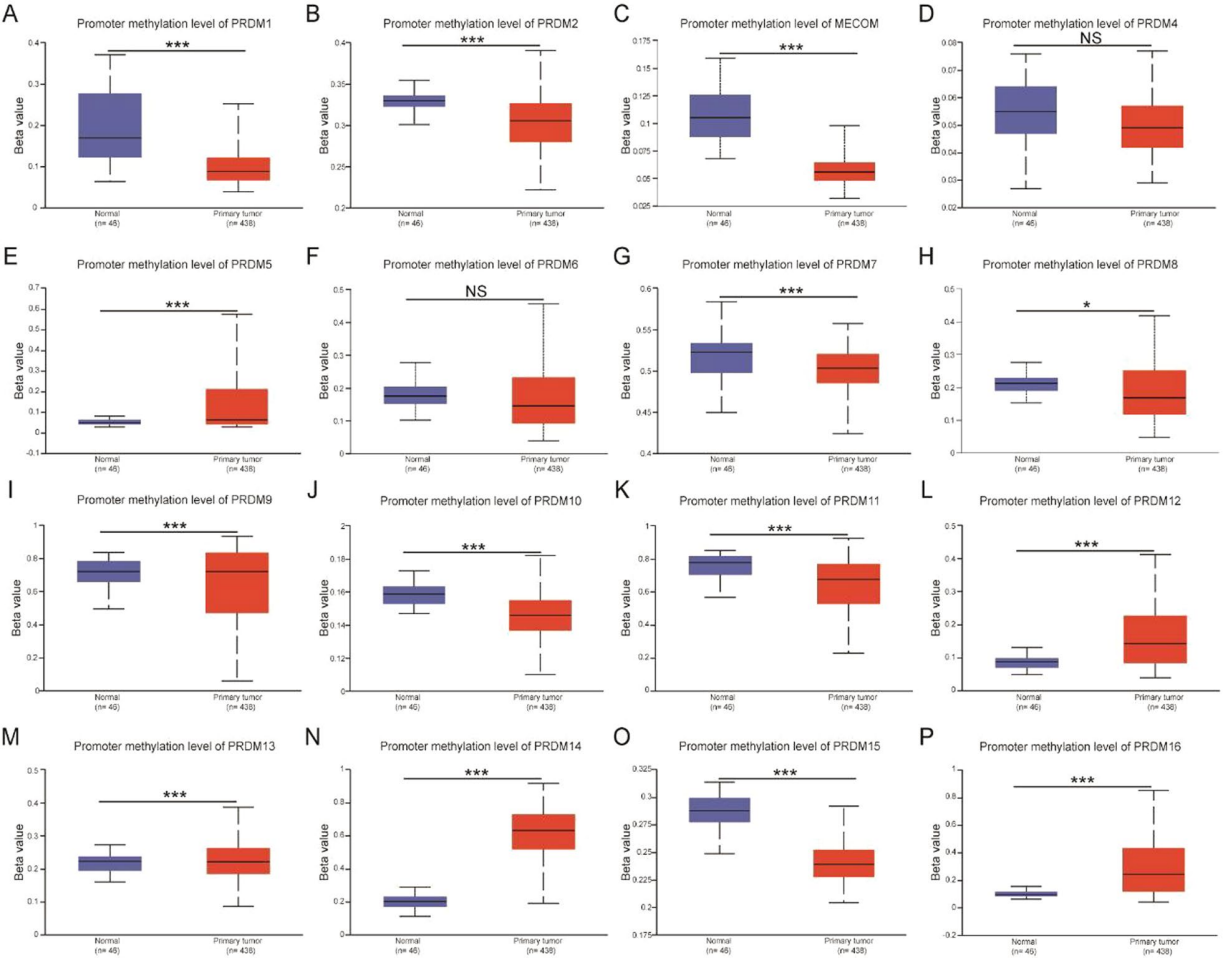
The correlation between the methylation status of PRDM family members and patients with UCEC (UALCAN). Higher methylation levels are observed in PRDM1 **A**, PRDM2 **B**, MECOM **C**, PRDM7 **G**, PRDM10 **J**, PRDM11 K, PRDM15 O show low levels of methylation. PRDM5 E, PRDM8 H, PRDM9 I, PRDM12 **L**, PRDM13(M), PRDM14 N, and PRDM16 **P exhibit higher levels of methylation**, indicating their potential involvement in tumor development through epigenetic silencing. Conversely, no significant differences in methylation are found in PRDM4 **D** and PRDM6 **F**, suggesting their distinct roles in cancer progression. Statistical significance is represented by an asterisk (*p < 0.05, **p < 0.01, ***p < 0.001, NS: not significant), with a higher significance level indicating a more pronounced difference in methylation between normal and cancerous tissues

Figure 4A–P demonstrates that in normal tissues, PRDM1, PRDM2, MECOM, PRDM7, PRDM8, PRDM10, PRDM11, PRDM13, and PRDM15 genes exhibit higher promoter methylation frequencies compared to UCEC patients. Conversely, PRDM5, PRDM9, PRDM12, PRDM14, and PRDM16 genes show lower promoter methylation in normal tissues compared to UCEC tissues. These results suggest a correlation between the methylation status of MECOM, PRDM5, PRDM7, PRDM12, PRDM15, and PRDM16 genes and their mRNA expression levels. There is a correlation between the methylation status of PRDM genes and their mRNA expression levels. Methylation is an epigenetic modification, and when a gene's promoter region is highly methylated, transcription initiation is hindered, leading to decreased mRNA expression levels. Studies have shown that the PRDM2, PRDM5, and PRDM16 promoters in lung cancer cells are methylated, resulting in suppressed expression (Tan et al. 2014). This study indicates that the methylation status of PRDM gene family members can serve as a regulatory mechanism for gene expression, where increased methylation levels are typically associated with reduced mRNA expression. However, the mRNA expression levels of PRDM1, PRDM2, PRDM8, and PRDM11 were higher in normal and cancer tissues. This discrepancy in mRNA expression levels could be attributed to the influence of other biological pathways.

Subsequently, we further explored the correlation between the methylation status of PRDM genes and various clinicopathological parameters, including patient weight, tumor histology, individual cancer stage, and tumor grade. Figure S4 demonstrates a significant association between the methylation status of PRDM genes (excluding PRDM4, PRDM6, and PRDM8) and patient weight. At the same time, we observe the promoter methylation levels of multiple members of the PRDM gene family across different types of uterine tissues. Specifically, these include comparisons of gene methylation levels in normal tissue, endometrioid carcinoma, serous carcinoma, and mixed-type (endometrioid and serous) carcinoma. Figure S5 shows that PRDM1, PRDM2, and MECOM genes exhibit highly significant differences in methylation levels between normal tissue and all types of cancer. Similarly, PRDM5, PRDM7, PRDM8, PRDM9, PRDM10, PRDM13, PRDM14, PRDM15, and PRDM16 also show significant or extremely significant differences in some types of cancer. However, the promoter methylation levels of PRDM3, PRDM4, PRDM6, PRDM11, and PRDM12 do not show significant differences across all cancer types. Furthermore, the methylation status of PRDM2, PRDM5, PRDM6, PRDM8, PRDM9, PRDM10, PRDM12, PRDM13, PRDM14, and PRDM16 showed associations with cancer stage in UCEC, while no significant differences were observed in the promoter methylation of PRDM1, MECOM, PRDM4, PRDM7, PRDM11, and PRDM15 (Figure S6). Similarly, in Figure S7, apart from PRDM7, PRDM11, and PRDM15, the methylation status of PRDM genes demonstrated a significant correlation with tumor grade. Overall, these results suggest the potential involvement of most PRDM genes in the pathogenesis and progression of UCEC, and a summary of these results can be found in Table 4.

**Table 4:** The correlation between the methylation status of PRDMs and the clinicopathological parameters of UCEC patients

	Patient’s weight	Tumor histology	Individual cancer stage	Tumor grade
PRDM1	C	NC	NC	C
PRDM2	C	C	C	C
MECOM	C	NC	NC	C
PRDM4	NC	NC	NC	C
PRDM5	C	C	C	C
PRDM6	NC	C	C	C
PRDM7	C	NC	NC	NC
PRDM8	NC	C	C	C
PRDM9	C	C	C	C
PRDM10	C	C	C	C
PRDM11	C	NC	NC	NC
PRDM12	C	C	C	C
PRDM13	C	C	C	C
PRDM14	C	C	C	C
PRDM15	C	C	NC	NC
PRDM16	C	C	C	C

C: stands for correlation; NC: stands for no correlation

### GSEA analysis of PRDM family members

Investigating the potential mechanisms of PRDMs in uterine corpus UCEC) involved conducting a GSEA analysis using the LinkedOmics database to obtain information on biological pathways. Two significant biological pathways were identified, as shown in Table 5. The GSEA analysis revealed that members of the PRDM family were primarily enriched in pathways such as “ECM-receptor interaction,” “Hippo signaling pathway,” “TGF-beta signaling pathway,” “DNA replication,” and “metabolic pathways.” Interestingly, these pathways align with the impact of PRDMs on clinical pathological parameters. These findings provide valuable insights into the potential mechanisms of PRDMs in UCEC, underscoring their significance in tumor development and various biological processes.

**Table 5:** The KEGG Pathway of PRDMs in UCEC (LinkedOmics)

PRDMs	Enriched KEGG Pathway	Size	ES	NES	*P*-value	FDR
PRDM1	ECM-receptor interaction	80	0.70	2.13	0	0
	Malaria	46	0.68	1.92	0	0.000
PRDM2	Circadian rhythm	30	0.66	1.69	0.006	0.024
	Hippo signaling pathway	25	0.64	1.58	0.015	0.052
PRDM4	TGF-beta signaling pathway	83	0.63	1.80	0	0.004
	Hedgehog signaling pathway	44	0.62	1.62	0.0025	0.054
PRDM5	Hedgehog signaling pathway	44	0.64	1.74	0	0.009
	Fanconi anemia pathway	44	0.58	1.55	0	0.279
PRDM7	Nitrogen metabolism	17	0.58	1.45	0.05	0.640
	Basal transcription factors	44	0.56	1.64	0	0.301
PRDM9	Nicotine addiction	40	0.69	1.75	0	0.023
	Olfactory transduction	259	0.61	1.69	0	0.030
PRDM10	Circadian rhythm	30	0.65	1.49	0.026	0.150
	Fanconi anemia pathway	44	0.63	1.52	0.007	0.159
PRDM11	Hedgehog signaling pathway	44	0.62	1.79	0	0.028
	Nicotine addiction	40	0.59	1.67	0	0.046
PRDM12	DNA replication	36	0.73	2.121	0	0
	One carbon pool by folate	18	0.68	1.678	0.011	0.022
PRDM13	Ribosome	130	0.64	2.201	0	0
	RNA polymerase	31	0.62	1.76	0.003	0.012
PRDM14	Caffeine metabolism	5	0.86	1.51	0.017	0.141
	Histidine metabolism	21	0.68	1.637	0.009	0.122
PRDM15	Primary immunodeficiency	36	0.53	1.453	0.027	0.757
	Taste transduction	80	0.51	1.546	0.010	0.773
PRDM16	Type II diabetes mellitus	45	0.68	1.857	0	0.008
	GABAergic synapse	88	0.67	2.017	0	0.005

ES: enrichment score, NES: normalized enrichment score, FDR: false discovery rate

### Genetic variation, interaction analysis, and neighbor gene network of PRDMs in UCEC patients

This study aimed to explore the genetic variations, correlations, and networks of PRDM family members in UCEC patients. We used the cBioPortal online tool to achieve this and analyzed the TCGA_UCEC cohort, which consisted of 527 UCEC patients.

**Fig. 5:**
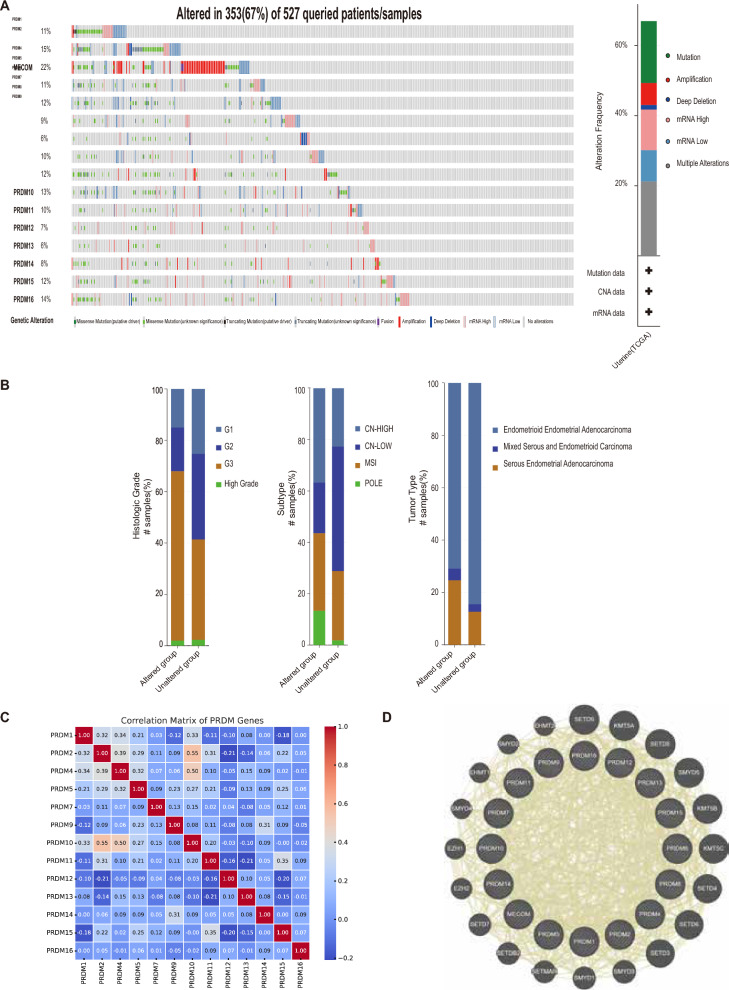
Gene alterations, interaction analysis, and neighboring gene network of different PRDMs in UCEC patients. **A** The changes in expression levels of different members of the PRDM family were analyzed in UCEC using data from cBioPortal. **B** A bar graph was generated based on data from cBioPortal to illustrate the distribution and types of PRDM gene mutations in different subgroups of UCEC. **C** The interrelationships between PRDM gene expressions in UCEC were visualized using a correlation coefficient heatmap, which was generated using data from cBioPortal. **D** The gene network generated from GeneMANIA revealed the presence of PRDM among the top 20 most closely related genes, indicating its close association with neighboring genes

Among the patients included in our analysis, we observed genetic variations in PRDMs in 353 samples, resulting in a mutation rate of 67% (Fig. 5A). Specifically, we found that MECOM, PRDM2, PRDM16, and PRDM10 were the four genes with the highest genetic variations. These variations included amplification, deep deletion, high mRNA expression, and missense mutations, with mutation rates of 22%, 15%, 14%, and 13%, respectively (Fig. 5A).

To further investigate the PRDM gene family's mutation status in different UCEC subgroups, we examined the mutation profiles in Fig. 5B. Additionally, we explored the potential co-expression relationships between PRDMs by analyzing the mRNA expression of UCEC using the cBioPortal database (RNA Seq V2 RSEM). Our analysis revealed a high correlation between PRDM2, PRDM4, and PRDM10, while the correlations between PRDM5, PRDM11, PRDM12, PRDM13, and PRDM15 were low to moderate (Fig. 5C). The figure shows strong positive correlations between some gene pairs, such as PRDM2 and PRDM10 (correlation coefficient 0.55) and PRDM4 and PRDM10 (correlation coefficient 0.50). Additionally, some negative correlations can be observed between gene pairs, such as PRDM2 and PRDM12 (− 0.21) and PRDM15 and PRDM12 (− 0.20), which may suggest the phenomenon of compensatory expression (Fig. 5C).

To gain insights into the interactions between PRDMs, we constructed a protein–protein interaction network using GeneMANIA. The network (Fig. 5D) highlighted the close associations between the PRDM gene family and other genes such as SETD9, KMT5A, SETD5, SMYD5, KMT5B, KMT5C, SETD4, SETD6, SETD3, SMYD3, SMYD1, SETMAR, SETDB2, SETD7, EZH2, EZH1, SMYD4, EHMT1, SMYD2, and EHMT2.

These findings provide valuable insights into the genetic variations, interactions, and neighbor gene networks of PRDMs in UCEC patients. This knowledge contributes to a deeper understanding of the roles and relationships of PRDMs in UCEC.

### Immune cell infiltration of PRDMs in UCEC patients

**Fig. 6:**
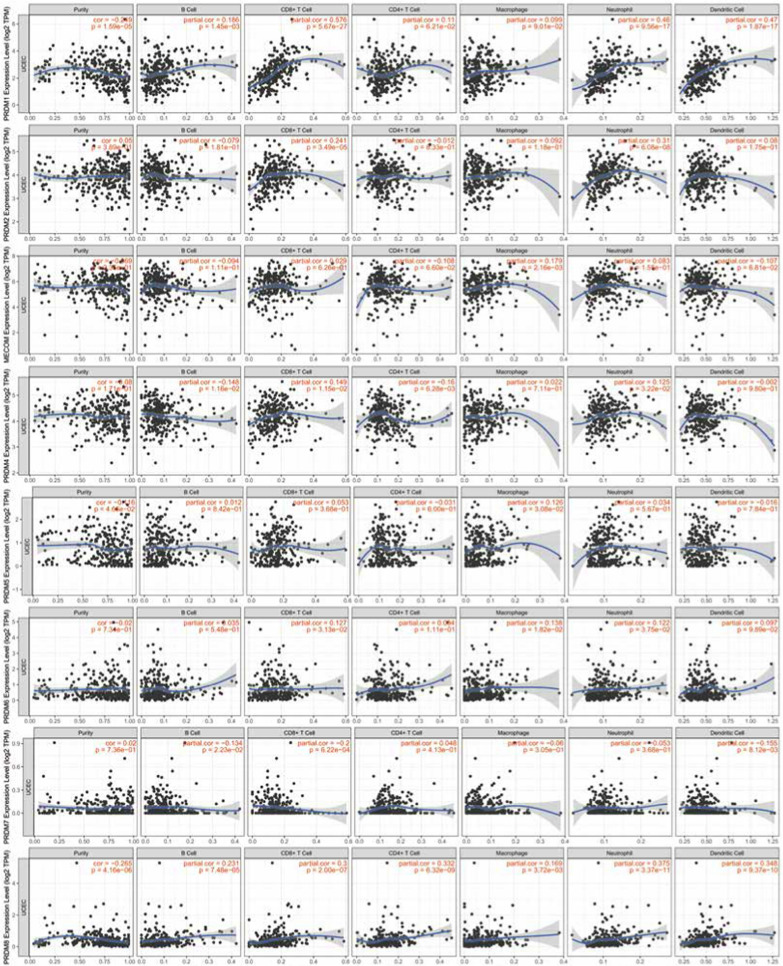

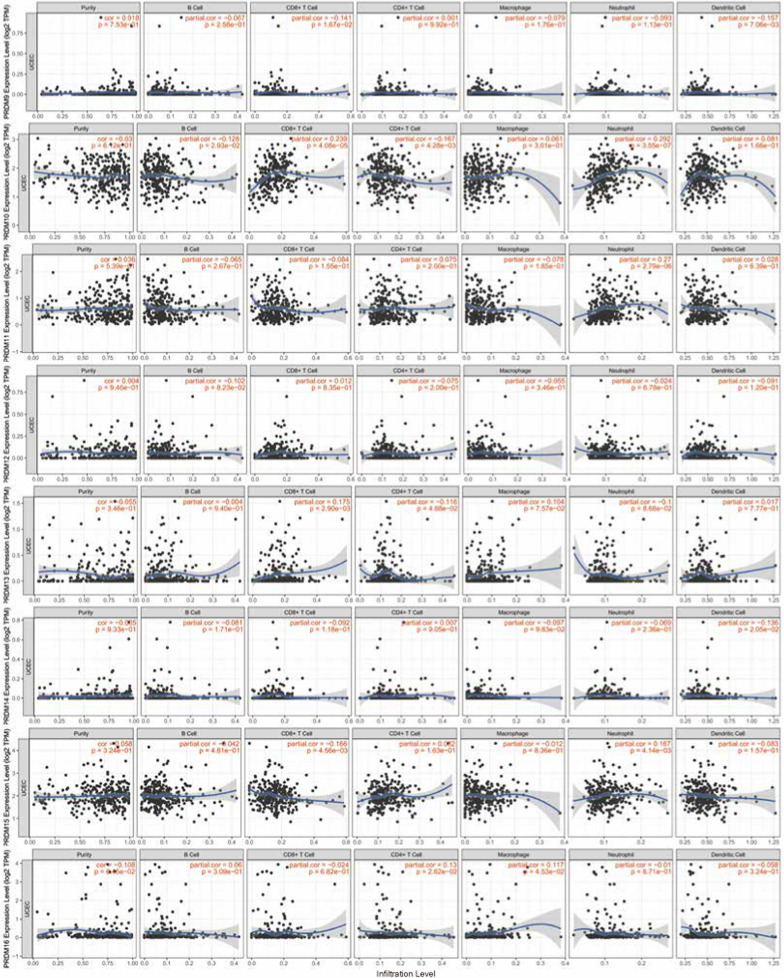
Correlation between PRDM genes and immune infiltration in UCEC (TIMER2.0). Each figure illustrates the level of correlation between PRDM genes and various immune cell types. This includes B cells, CD8 + T cells, CD4 + T cells, immune cells associated with PRDM10 and PRDM11, and PRDM2, PRDM2, PRDM8, PRDM8, neutrophils, and dendritic cells. The strength and statistical significance of these correlations are represented by the size and color intensity of the dots in each figure

The infiltration status of immune cells is crucial for determining cancer prognosis, including uterine corpus endometrial carcinoma (UCEC). Our study used the TIMER database to analyze the relationship between the PRDM gene family and immune cell infiltration in UCEC patients. Our findings, depicted in Fig. 6, indicate a positive correlation between the mRNA expression of PRDM1, PRDM2, PRDM8, PRDM10, and PRDM11 with the infiltration of B cells, CD8 T cells, CD4 T cells, neutrophils, and dendritic cells. However, in UCEC patients, there were no significant correlations observed between the expression of MECOM, PRDM4, PRDM5, PRDM6, PRDM7, PRDM9, PRDM12, PRDM13, PRDM14, PRDM15, PRDM16, PRDM17 and the level of immune cell infiltration.

To account for confounding factors, we performed multivariate analysis with corrections for age and race. Even after adjusting for these variables, CD8 T cells, neutrophils, PRDM5, PRDM7, and PRDM12 remained significantly associated with the survival of UCEC patients, as shown in Table 6.

**Table 6:** Clinical relevance of tumor immune subsets in a multivariable Cox proportional hazard model (TIMER 2.0)

	Coef	HR	95% CI (lower)	95% CI (upper)	*p*-value
Purity	1.59	4.90E + 00	0.870	2.76E + 01	0.072
Age	0.01	1.01E + 00	0.975	1.05E + 00	0.505
race Black	0.16	1.17E + 00	0.133	1.03E + 01	0.885
race White	– 0.05	9.50E-01	0.113	7.97E + 00	0.962
B cell	– 10.32	0.00E + 00	0.000	1.87E + 00	0.065
CD8 T cell	– 16.57	0.00E + 00	0.000	1.00E-03	0.001
CD4 T cell	– 7.88	0.00E + 00	0.000	5.19E + 01	0.192
Macrophage	2.69	1.48E + 01	0.003	8.50E + 04	0.542
Neutrophil	19.07	1.92E + 08	32.376	1.14E + 15	0.017
Dendritic	3.72	4.14E + 01	0.182	9.44E + 03	0.179
PRDM1	0.32	1.38E + 00	0.784	2.41E + 00	0.267
PRDM2	0.80	2.22E + 00	0.941	5.25E + 00	0.069
MECOM	0.53	1.69E + 00	0.939	3.04E + 00	0.080
PRDM4	– 0.70	4.98E-01	0.177	1.41E + 00	0.188
PRDM5	0.86	2.37E + 00	1.256	4.48E + 00	0.008
PRDM6	0.22	1.24E + 00	0.833	1.85E + 00	0.289
PRDM7	– 6.90	1.00E-03	0.000	2.22E-01	0.012
PRDM8	– 0.40	6.73E-01	0.288	1.57E + 00	0.360
PRDM9	1.55	4.73E + 00	0.337	6.63E + 01	0.249
PRDM10	– 1.14	3.19E-01	0.093	1.10E + 00	0.070
PRDM11	0.06	1.06E + 00	0.418	2.71E + 00	0.897
PRDM12	9.75	1.72E + 04	309.236	9.55E + 05	0.000
PRDM13	0.45	1.57E + 00	0.257	9.58E + 00	0.625
PRDM14	– 3.72	2.40E-02	0.000	1.70E + 00	0.086
PRDM15	0.05	1.05E + 00	0.369	2.99E + 00	0.927
PRDM16	– 0.14	8.69E-01	0.475	1.59E + 00	0.649

UCEC: uterine corpus endometrial cancer; Coef: regression coefficient; HR: hazard ratio

These results provide valuable insights into the association between the PRDM gene family and immune cell infiltration in UCEC patients, suggesting their potential role in regulating the tumor immune microenvironment.

### Study on the role of MECOM in uterine endometrial *cancer* cells and its impact on cell proliferation, apoptosis, and migration

Through bioinformatics analysis, it was discovered that the expression of MECOM in UCEC (Uterine Corpus Endometrial Carcinoma) tissues is significantly higher than in normal tissues, suggesting its potential key role in tumor development and progression. Additionally, the methylation status of MECOM correlates with its mRNA expression level, indicating that its expression may be subject to epigenetic regulation. Despite no significant correlation between MECOM and immune cell infiltration, its high expression in cancer tissues may imply a potential role in modulating the tumor microenvironment, particularly in tumor immune evasion. Furthermore, MECOM is one of the genes with the highest genetic variation, including amplification, deep deletion, high mRNA expression, and missense mutations, which may indicate its key role in tumor development. Further in vitro cell experiments will be conducted to validate the results of bioinformatics analysis.

**Fig. 7:**
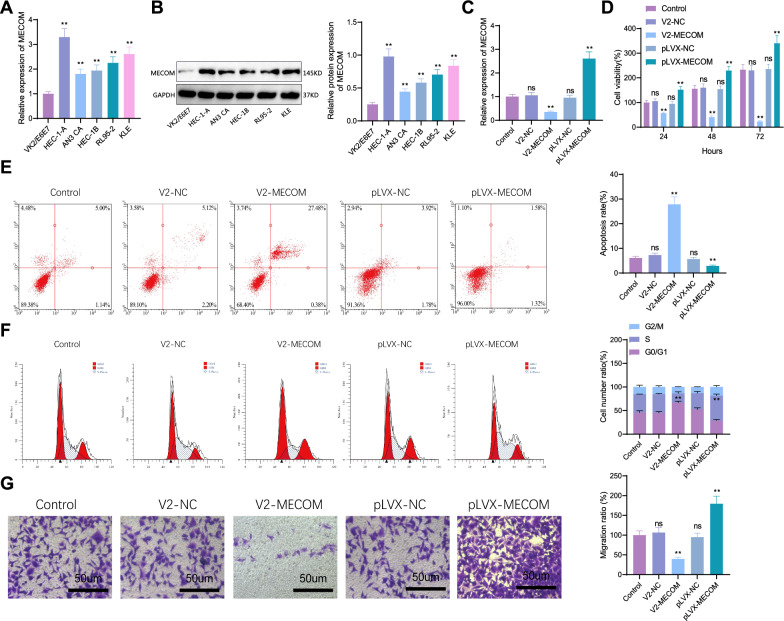
Expression of MECOM in Uterine Endometrial Cancer and Normal Cervical Epithelial Cells and Its Impact on Cellular Functions. **A** Expression levels of MECOM mRNA in different cell lines (compared to the Control group, ****p < 0.0001; ns: not significant); **B** Expression levels of MECOM protein in different cell lines (compared to the Control group, ****p < 0.0001; ***p < 0.001; **p < 0.01; ns: no statistical significance); **C** Impact of overexpression and knockout of MECOM gene on mRNA levels. (Compared to the Control group, ****p < 0.0001; ns: not significant) **D** Impact of overexpression and knockout of MECOM gene on cell proliferation. (Compared to the Control group, ****p < 0.0001; ***p < 0.001; **p < 0.01; ns: no statistical significance); **E** Analysis of cell apoptosis rate (Compared to the Control group, ****p < 0.0001; ***p < 0.001; ns: no statistical significance); **F** Analysis of cell cycle changes (Compared to the Control group, ****p < 0.0001; ***p < 0.001; ns: no statistical significance); **G** Transwell cell migration experiment (Compared to the Control group, ****p < 0.0001; ns: not significant), all cell experiments repeated three times

In this study, several uterine endometrial cancer cell lines (HEC-1-A, AN3 CA, HEC-1B, RL95-2, KLE) and a normal cervical epithelial cell line VK2/E6E7 were chosen to investigate the expression of MECOM. RT-PCR results showed that both MECOM mRNA (Fig. 7A) and protein expression levels (Fig. 7B) were significantly higher in the uterine endometrial cancer cell lines compared to the normal epithelial cell line, with the highest expression level in the HEC-1-A cell line. Therefore, this cell line was selected for subsequent experiments. We also compared the expression levels of PRDMs in the normal cervical epithelial cell line VK2/E6E7 and the endometrial cancer cell line HEC-1-A. RT-PCR results showed that MECOM, PRDM7, and PRDM15 transcript levels were significantly higher in HEC-1-A cells than in VK2/E6E7 cells. In contrast, the mRNA expression levels of PRDM1, PRDM2, PRDM4, PRDM5, PRDM6, PRDM8, PRDM11, PRDM12, and PRDM16 were significantly lower in HEC-1-A cells compared to VK2/E6E7 cells, consistent with our UALCAN analysis results (Figure S8).

Furthermore, we verified the overexpression and knockout of the MECOM gene. RT-qPCR results (Fig. 7C) showed that in the MECOM overexpression experiment, MECOM gene expression levels in the HEC-1-A cells of the pLVX-MECOM group significantly increased, doubling in expression compared to the blank control group, while the pLVX-NC group showed normal expression. Meanwhile, MECOM knockout HEC-1-A cell lines were successfully constructed using CRISPR/Cas9 technology, showing a significant reduction in MECOM gene expression, fourfold lower than the blank control group, with a marked difference, while the V2-NC group showed no difference. CCK-8 assay results (Fig. 7D) demonstrated that in the MECOM overexpression experiment, the proliferation ability of HEC-1-A cells in the pLVX-MECOM group was significantly enhanced compared to the pLVX-NC group, with a noticeable promotion in cell growth rate. Conversely, in cells with MECOM knocked out, cell proliferation ability was significantly reduced, and the cell growth rate was notably inhibited. Flow cytometry (Fig. 7E) revealed that the apoptosis rate of HEC-1-A cells in the pLVX-MECOM group was significantly reduced, whereas in cells with MECOM knocked out, the apoptosis rate increased. MECOM may inhibit pathways leading to apoptosis, and its overexpression might enhance these pathways, thereby reducing the rate of cell apoptosis, while its absence might lead to the inhibition of these apoptosis pathways. Flow cytometry further analyzed changes in the cell cycle (Fig. 7F). Compared to the control group, a significant difference in the proportion of S-phase cells was observed in the cell cycle distribution of both the MECOM overexpression and knockout groups. Overexpression of MECOM significantly increased the proportion of S-phase cells, while knockout of MECOM significantly reduced it. It indicates that MECOM plays an important role in regulating the cell cycle and promoting cell entry into the S phase, and it may be related to the enhanced ability of cell proliferation. Transwell migration assay results (Fig. 7G) showed that compared to the control group, the migration ability of cells in the MECOM overexpression group was significantly enhanced, possibly due to MECOM overexpression promoting tumor cell invasion and metastasis. Conversely, cell migration ability was significantly reduced in cells with MECOM knocked out, inhibiting tumor cell invasion and metastasis.

In summary, the overexpression of MECOM in HEC-1-A uterine endometrial cancer cells promoted cell proliferation, cell cycle progression, and cell migration ability while inhibiting cell apoptosis. The knockout of MECOM had the opposite effect. These comprehensive results reveal the important role of MECOM in uterine endometrial cancer cells, providing crucial clues for further investigation into its mechanism and potential treatment strategies.

### MECOM regulates cell cycle and apoptosis through the TGF-*beta* signaling pathway

**Fig. 8:**
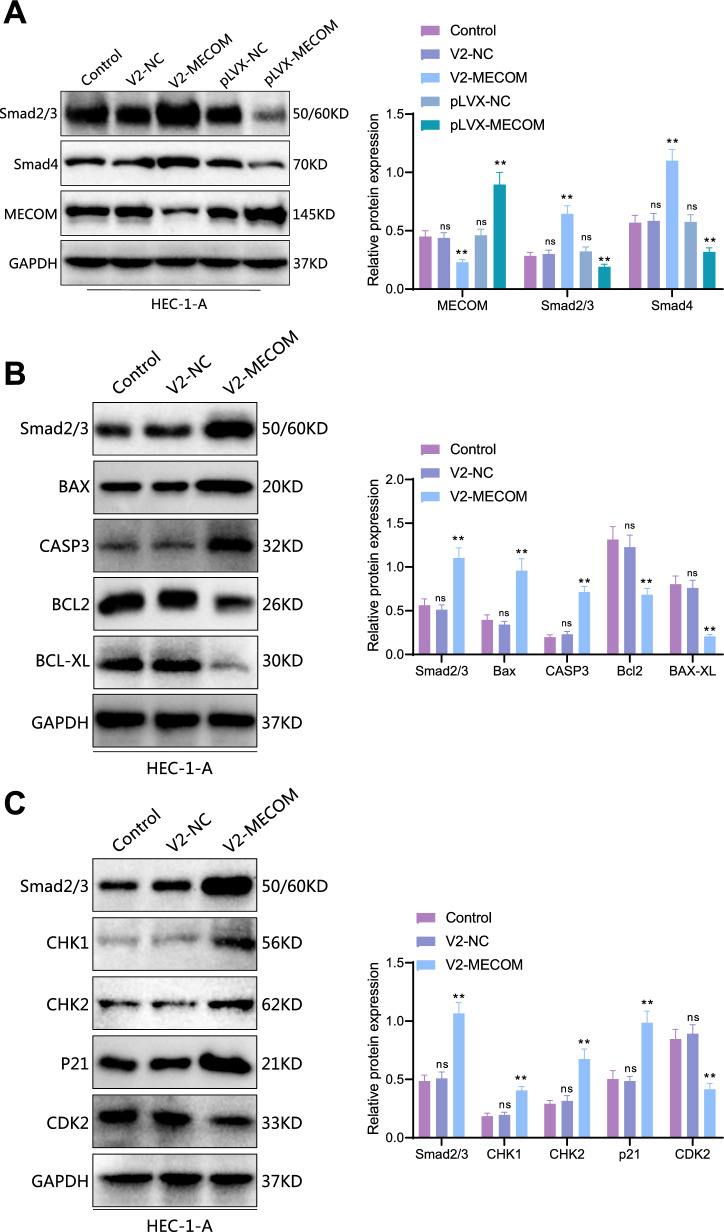
Impact of MECOM Regulation of Smad Proteins on Apoptosis and Cell Cycle Regulation in HEC-1-A Cells. **A** Western blot analysis of changes in Smad protein levels after MECOM knockout and overexpression. The left image shows the changes in Smad protein levels in HEC-1-A cells after MECOM knockout and overexpression. The right chart shows the quantitative analysis (Compared to the Control group, ****p < 0.0001; ns: insignificant). **B** Western blot analysis of the impact of MECOM knockout on apoptosis-related proteins. The left image shows the protein expression levels of BAX, caspase-3 (CASP3), Bcl-2, and Bcl-xL in the control group (Control), empty vector control group (V2-NC), and MECOM knockout (V2-MECOM). The right chart is the quantitative analysis of the corresponding proteins, showing a significant increase in the expression of Smad2/3, BAX, and caspase-3 in cells with MECOM knockout, while the expression of Bcl-2, Bcl-xL significantly reduced (Compared to the Control group, ****p < 0.0001; ns: not significant). **C** Western blot analysis of the impact of MECOM knockout on cell cycle regulatory proteins. The left image shows the protein expression levels of CHK1, CHK2, p21, and CDK2 in the control group (Control), empty vector control group (V2-NC), and MECOM knockout (V2-MECOM). The right chart is the quantitative analysis of the corresponding proteins, showing a significant increase in the expression of Smad2/3, CHK1, CHK2, and p21 in cells with MECOM knockout, while the expression of CDK2 significantly reduced (Compared to the Control group, ****p < 0.0001; ns: not significant). All cell experiments were repeated three times

GSEA analysis shows that MECOM is associated with the TGF-beta signaling pathway. The TGF-beta signaling pathway is vital in developing various cancers, particularly cell proliferation, differentiation, and apoptosis (Cheng et al. 2018; Lin et al. 2021). Exploring how MECOM influences this pathway may reveal new therapeutic targets or pathological mechanisms. In this study, we conducted a Western blot experiment to delve into the biological function of MECOM in the uterine endometrial cancer HEC-1-A cell line. As shown in Fig. 8A, in HEC-1-A cells with MECOM overexpression, key factors Smad2/3 and Smad4 in the TGF-beta signaling pathway were significantly reduced compared to the control group. Conversely, Smad proteins were significantly increased in cells with MECOM knocked out compared to the control. In the TGF-beta signaling pathway, Smad proteins play a crucial role and are vitally important in regulating cell proliferation and apoptosis.

Based on the results of preliminary cell function experiments, we hypothesized that MECOM might regulate the cell cycle and apoptosis process by affecting Smad proteins. Furthermore, we explored the specific molecular mechanisms through which it regulates apoptosis and the cell cycle. As depicted in Fig. 8B, compared to the control group, the expression levels of apoptosis-promoting proteins BAX and caspase-3 were significantly increased in HEC-1-A cells with MECOM knocked out, while the expression of anti-apoptotic proteins Bcl-2 and Bcl-xL was significantly reduced. A similar trend was observed in Fig. 8C, where the expression levels of cell cycle inhibitory proteins CHK1, CHK2, and p21 were significantly elevated in cells with MECOM knocked out, while the expression level of CDK2 was significantly reduced. Thus, we speculate that MECOM might regulate the activity of Smad, thereby affecting the blockage of the S phase of the cell cycle and the occurrence of cell apoptosis.

In summary, our study emphasizes the potential impact of MECOM in the development of uterine endometrial cancer, revealing its significant influence on the proliferation, survival, and death of tumor cells through its effect on key proteins Smad. These findings provide a strong scientific basis for future targeted therapeutic strategies against MECOM.

## Discussion

This study delves deeply into the role of the PRDM family in uterine endometrial cancer (UCEC), particularly MECOM. Compared to previous studies, our findings reveal the significant role of PRDM family members in tumor formation, cell proliferation, apoptosis, and the maintenance of the immune microenvironment (Eroglu et al. 2014; Fog et al. 2015; Casamassimi et al., 2020; Rienzo et al. 2021a). Our research further focuses on the impact of MECOM on the TGF-beta signaling pathway, a topic not extensively explored in earlier studies. Our results suggest that this interaction may play a key role in the pathogenesis of UCEC, offering a new perspective on the role of the PRDM family in cancer biology.

In our study, we discovered significant differences in the expression patterns and methylation states of PRDM family members in uterine endometrial carcinoma (UCEC) compared to normal tissues, aligning with previous research findings (Sorrentino et al. 2018; Faure et al. 2020; Fan et al. 2015). Further, we explored the correlation between these differences and the clinical pathological characteristics of UCEC, revealing a close association between the expression changes of specific PRDM family members and the severity of the disease and patient prognosis. It provides new insights into the role of the PRDM family in UCEC and suggests potential biomarkers for future clinical prognosis assessment.

Further exploring the function of MECOM in the HEC-1-A cell line, we found that overexpression of MECOM significantly inhibited cell proliferation and migration and promoted apoptosis. It is consistent with the roles of PRDM family members in tumor cell proliferation and apoptosis reported in other studies (Nishikawa et al. 2007; Rienzo et al. 2021b). However, the uniqueness of our study lies in the fact that we are the first to reveal that MECOM influences the key proteins of the TGF-beta signaling pathway, specifically Smad proteins. We hypothesize that MECOM may regulate the transcription levels of Smad proteins by binding to their gene promoter regions or by interacting with Smad proteins to affect their activity, localization, or stability. It, in turn, influences TGF-beta signaling, leading to effects on cell cycle S-phase arrest and apoptosis, thereby impacting endometrial cancer (UCEC). It provides a new mechanistic understanding of MECOM's role in regulating the cell cycle and apoptosis.

In our research, we found that in uterine endometrial carcinoma (UCEC), overexpression of MECOM enhances cell proliferation and migration, while its knockout suppresses these processes and promotes apoptosis. It aligns partially with observations of PRDM family members in other disease types (Zhou et al. 2019). It's important to note, however, that the tumor microenvironment, genetic variations, and the activity of signaling pathways in different cancer types can influence the specific actions of PRDM family members (Sorrentino et al. 2018; Casamassimi et al., 2020). Thus, while some similarities in PRDM family functions across various cancers exist, significant differences in their specific functions and mechanisms of action in different cancer contexts are likely. It implies that our observations of MECOM in UCEC, despite sharing similarities with findings in some cancers, require further investigation to understand the unique mechanisms of MECOM in regulating UCEC cell behavior and its varied roles across different cancer types.

Our study also explored the impact of MECOM on cell proliferation, migration, and apoptosis in UCEC (uterine endometrial carcinoma) cells. We discovered that overexpression of MECOM promotes cell proliferation and migration, while its knockout inhibits these biological processes and enhances apoptosis. This finding is consistent with observations of PRDM family members in various cancer contexts found in other studies (Sorrentino et al. 2018; Casamassimi et al., 2020). However, our research further reveals the unique mechanisms by which MECOM regulates cell behavior in UCEC, deepening the understanding of its role in this specific cancer type. Reports on the role of PRDM in UCEC are limited. However, PRDM family members have been shown to influence various biological behaviors of tumors, such as proliferation, invasion, migration, and apoptosis, either by promoting or inhibiting tumor development in different cancers. The aberrant expression of PRDM family members is associated with disease prognosis in various cancers, suggesting their potential as diagnostic markers and therapeutic targets (Casamassimi et al., 2020). Nevertheless, the same PRDM family member may have different roles in different types of cancer, and different PRDM family members may influence tumor development by regulating different genes and signaling pathways, depending on the specific cancer type and microenvironment (Di Donato et al. 2023).

Our study highlights the genetic changes in the PRDM family, especially frequent genetic alterations in genes like MECOM, PRDM2, PRDM16, and PRDM10. These genetic changes may play a key role in the development and progression of UCEC. Our findings offer a new perspective on understanding the role of the TGF-beta signaling pathway in the onset and progression of uterine endometrial cancer.

The study provides new insights into the treatment and prognosis assessment of uterine endometrial cancer (UCEC). The expression patterns and methylation status of the PRDM family, particularly MECOM in UCEC, lay the groundwork for identifying new biomarkers and potential therapeutic targets. For example, the upregulation and abnormal methylation of MECOM found in UCEC may hint at its potential value in early disease diagnosis. Moreover, the interaction of MECOM with the TGF-beta signaling pathway reveals new therapeutic mechanisms, potentially aiding in the development of targeted therapies against this pathway. However, discovering the correlation between the expression of PRDM family members and the immune microenvironment in uterine endometrial cancer opens new avenues for designing immunotherapy strategies, especially in personalized medicine and precision treatment.

Despite providing new insights into the role of the PRDM family in UCEC, our study has limitations. First, it relies on data from public databases, which may have inconsistencies and quality issues. Second, our conclusions may need validation in a broader patient population due to the lack of large-scale clinical samples and prospective studies. We have not yet determined how MECOM influences the TGF-beta signaling pathway and UCEC through its effects on Smad proteins. In future experiments, we can first verify how MECOM affects Smad proteins. It can be done by using RNA-seq or microarray analysis to examine the expression profiles of Smad-related genes under different levels of MECOM expression, identifying potential downstream targets.Furthermore, we can establish xenograft tumor models in nude mice to evaluate the impact of MECOM on UCEC growth. While we explored the function of MECOM at the cellular level, these findings need further confirmation through in vivo experiments and clinical trials. Therefore, while our study offers valuable preliminary insights, more research is needed before these findings can be translated into clinical applications.

The TCGA project, through extensive sequencing and analysis of numerous cancer samples, has unveiled the molecular landscape of various cancers, including UCEC. In UCEC, TCGA identified several key molecular subtypes: POLE-ultramutated, microsatellite instability (MSI), p53-mutant, and no specific molecular profile (NSMP). These subtypes reflect the genetic heterogeneity of tumors and are closely associated with clinical phenotypes, treatment responses, and prognosis.

From a clinical perspective, integrating TCGA molecular subtyping with traditional prognostic factors (such as age, tumor grade, stage, lymph node status, etc.) can provide more precise patient risk stratification. For instance, a young patient may have a better overall physiological condition, but if her tumor is of the p53-mutant subtype, her prognosis may be worse than expected, necessitating a more aggressive treatment approach. Conversely, an older patient, even with a tumor classified as POLE-ultramutated, may have a better survival outlook due to favorable molecular characteristics despite facing higher risks from surgery and chemotherapy.

Moreover, as mentioned in updated guidelines, molecular biomarker testing can aid clinicians in making treatment decisions based on specific molecular features of the patient's tumor, opting for the most appropriate treatment regimen, such as targeted therapy or immunotherapy, rather than solely relying on traditional clinicopathological indicators.

In summary, combining the latest molecular biology discoveries with clinical practice can enhance our understanding and treatment of endometrial cancer, offering more personalized medical care for patients (Raffone et al. 2021, 2022).

Future research should focus on overcoming the current study’s limitations and further exploring the role of the PRDM family in UCEC and other cancers. Firstly, conducting larger-scale and multi-center studies is necessary to confirm the role of PRDM family members in uterine endometrial cancer and their potential clinical applications. Secondly, laboratory studies should aim to understand the exact role of PRDM family members, especially MECOM, in the pathogenesis of uterine endometrial cancer, including their interactions with other molecules and signaling pathways. Additionally, exploring novel therapeutic strategies based on PRDM family members, such as targeted and immunotherapies, will be an important direction for future research. Ultimately, these studies may provide new methods for the treatment and prognosis improvement of patients with uterine endometrial cancer.

## Conclusion

**Fig. 9:**
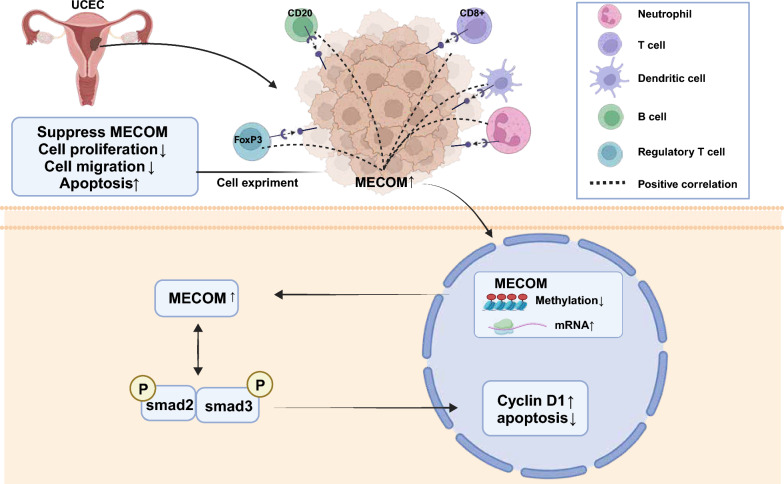
Abnormal expression of MECOM in uterine corpus endometrial carcinoma and its mechanistic involvement in regulating the Hippo signaling pathway and affecting cell cycle and apoptosis. In endometrial cancer, decreased methylation levels of MECOM lead to increased expression. MECOM regulates cell cycle and apoptosis in endometrial cancer by affecting the phosphorylation levels of crucial TGF-beta signaling proteins, Smad. Additionally, changes in MECOM expression influence cell migration, which may subsequently affect tumor invasion and metastasis

This study thoroughly investigates the expression patterns and potential biological functions of PRDM family members in uterine endometrial cancer (UCEC). Through analysis of online databases, we found significant differences in the mRNA and protein levels of several PRDM family genes in UCEC tissues compared to normal tissues, with upregulation of MECOM, PRDM7, and PRDM15, and downregulation of PRDM1 and PRDM2, among others. Further clinical correlation analysis indicates that the expression of PRDM family members is closely related to patients' cancer staging, weight, menopausal status, and prognosis. Notably, the mutation frequency of MECOM is the highest in the PRDM2 family. Promoter methylation analysis shows that the methylation status of MECOM differs between normal and cancerous tissues. In vitro cellular experiments further validate the oncogenic role of MECOM in uterine endometrial cancer cells, indicating its regulation of the cell cycle and apoptosis by influencing critical proteins of the TGF-beta signaling pathway. Changes in MECOM also affect the cell's migration ability, potentially impacting tumor invasion and metastasis (Fig. 9). Overall, PRDM family members, especially MECOM, may play a key role in the onset and development of uterine endometrial cancer, providing potential targets for future targeted therapy.

## Supplementary Information

Additional file 1

Additional file 2

Additional file 3

Additional file 4

Additional file 5

Additional file 6

Additional file 7

Additional file 8

Additional file 9

## Data Availability

All data generated or analyzed during this study are included in this article.
