# Supplementary material for: MECOM and the PRDM gene family in uterine endometrial cancer: bioinformatics and experimental insights into pathogenesis and therapeutic potentials
Source: Mol Med. 2024 Oct 28;30:190. doi: 10.1186/s10020-024-00946-0 (PMC11514642; doi:10.1186/s10020-024-00946-0)
Supplement: Supplementary file 1 — Additional file 1 [file 10020_2024_946_MOESM1_ESM.pdf]

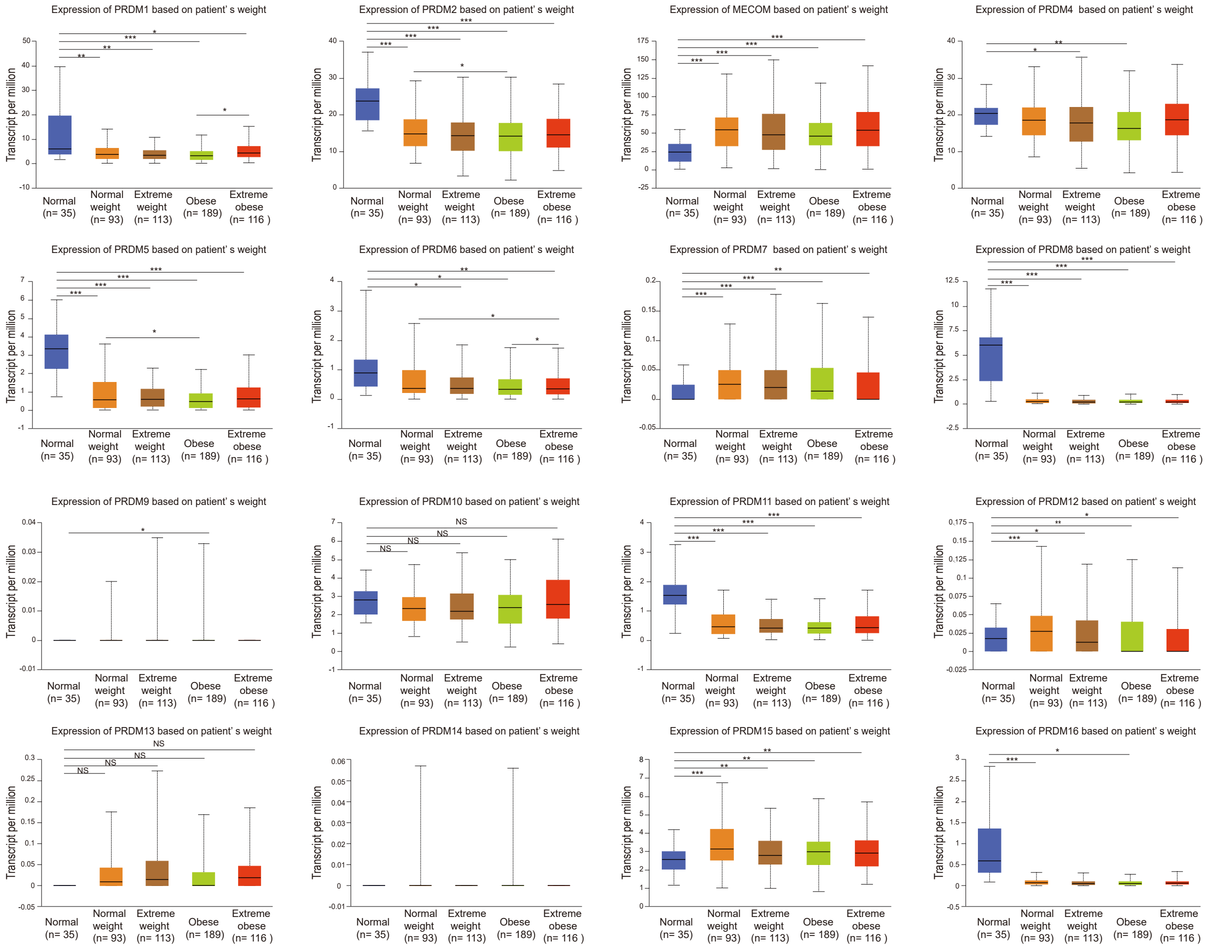

Expression of PRDM1 based on patient' s weight
Expression of PRDM2 based on patient' s weight
Expression of MECOM based on patient' s weight
Expression of PRDM4 based on patient' s weight
Expression of PRDM5 based on patient' s weight
Expression of PRDM6 based on patient' s weight
Expression of PRDM7 based on patient' s weight
Expression of PRDM8 based on patient' s weight
Expression of PRDM9 based on patient' s weight
Expression of PRDM10 based on patient' s weight
Expression of PRDM11 based on patient' s weight
Expression of PRDM12 based on patient' s weight
Expression of PRDM13 based on patient' s weight
Expression of PRDM14 based on patient' s weight
Expression of PRDM15 based on patient' s weight
Expression of PRDM16 based on patient' s weight
Transcript per million
Normal (n= 35)
Normal weight (n= 93)
Extreme weight (n= 113)
Obese (n= 189)
Extreme obese (n= 116 )
NS
