# Supplementary material for: MECOM and the PRDM gene family in uterine endometrial cancer: bioinformatics and experimental insights into pathogenesis and therapeutic potentials
Source: Mol Med. 2024 Oct 28;30:190. doi: 10.1186/s10020-024-00946-0 (PMC11514642; doi:10.1186/s10020-024-00946-0)
Supplement: Supplementary file 2 — Additional file 2 [file 10020_2024_946_MOESM2_ESM.pdf]

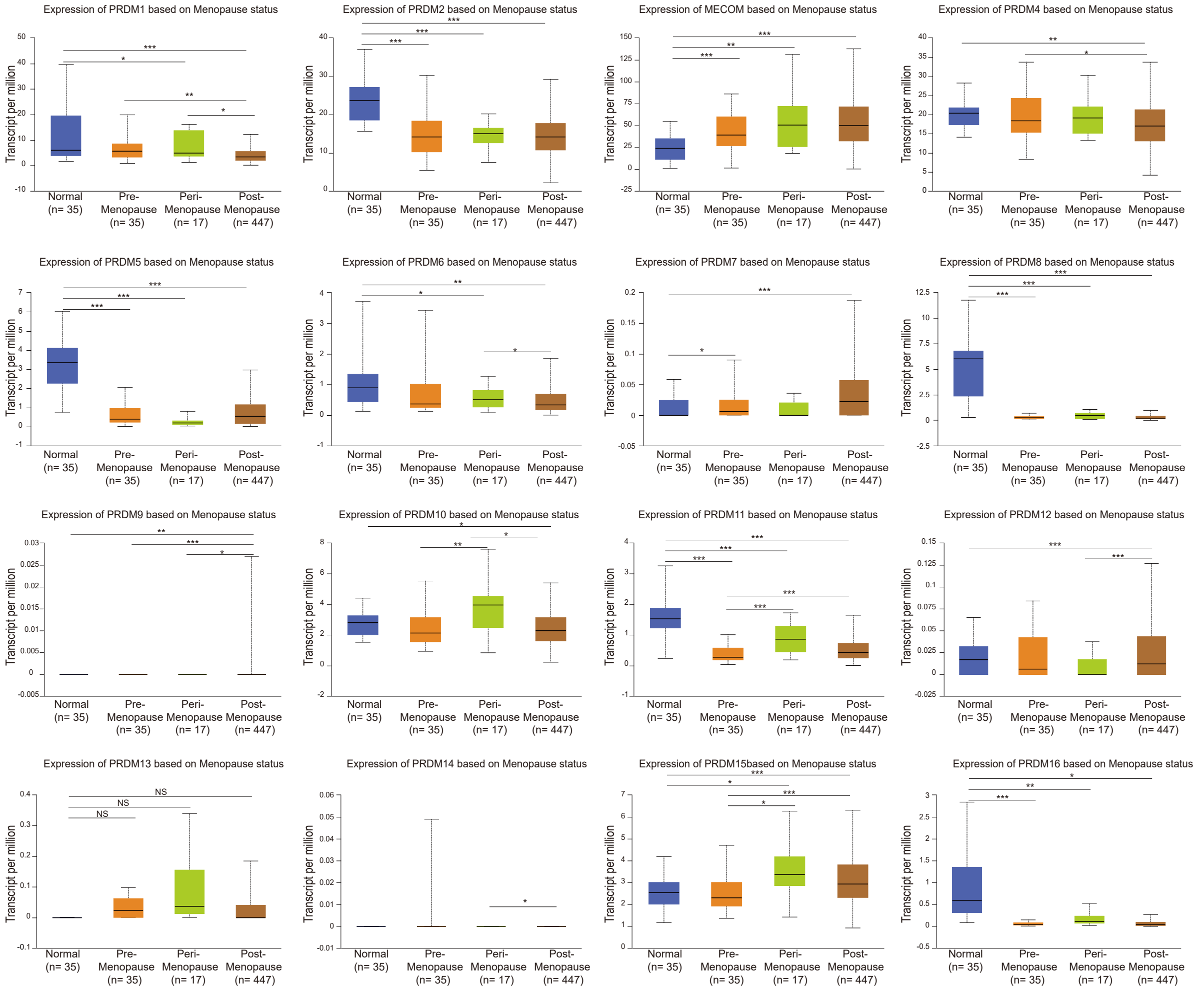
Expression of PRDM1 based on Menopause status
Expression of PRDM2 based on Menopause status
Expression of MECOM based on Menopause status
Expression of PRDM4 based on Menopause status
Expression of PRDM5 based on Menopause status
Expression of PRDM6 based on Menopause status
Expression of PRDM7 based on Menopause status
Expression of PRDM8 based on Menopause status
Expression of PRDM9 based on Menopause status
Expression of PRDM10 based on Menopause status
Expression of PRDM11 based on Menopause status
Expression of PRDM12 based on Menopause status
Expression of PRDM13 based on Menopause status
Expression of PRDM14 based on Menopause status
Expression of PRDM15based on Menopause status
Expression of PRDM16 based on Menopause status
Transcript per million
Normal (n= 35)
Pre-Menopause (n= 35)
Peri-Menopause (n= 17)
Post-Menopause (n= 447)
NS
