# Supplementary figures and images for: MECOM and the PRDM gene family in uterine endometrial cancer: bioinformatics and experimental insights into pathogenesis and therapeutic potentials
Source: Mol Med. 2024 Oct 28;30:190. doi: 10.1186/s10020-024-00946-0 (PMC11514642; doi:10.1186/s10020-024-00946-0)

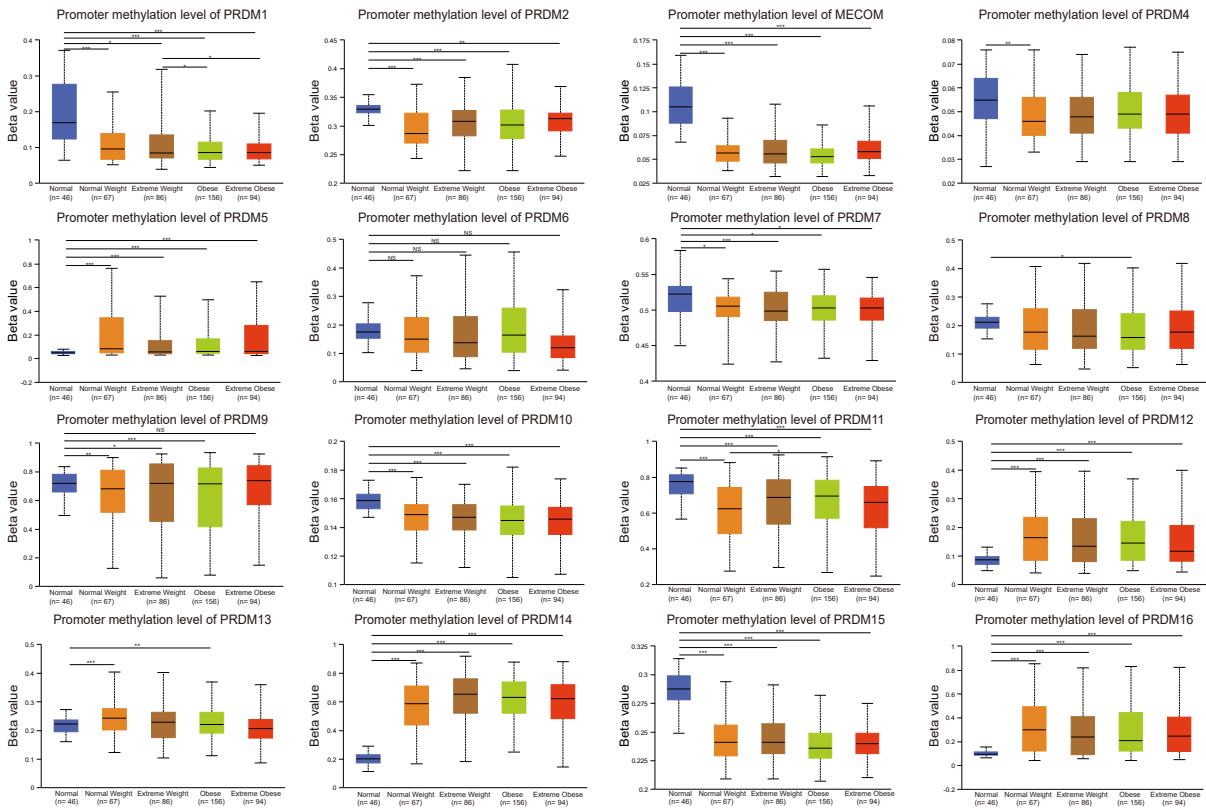

Supplement: Supplementary file 3 — Additional file 3 [file 10020_2024_946_MOESM3_ESM.pdf]

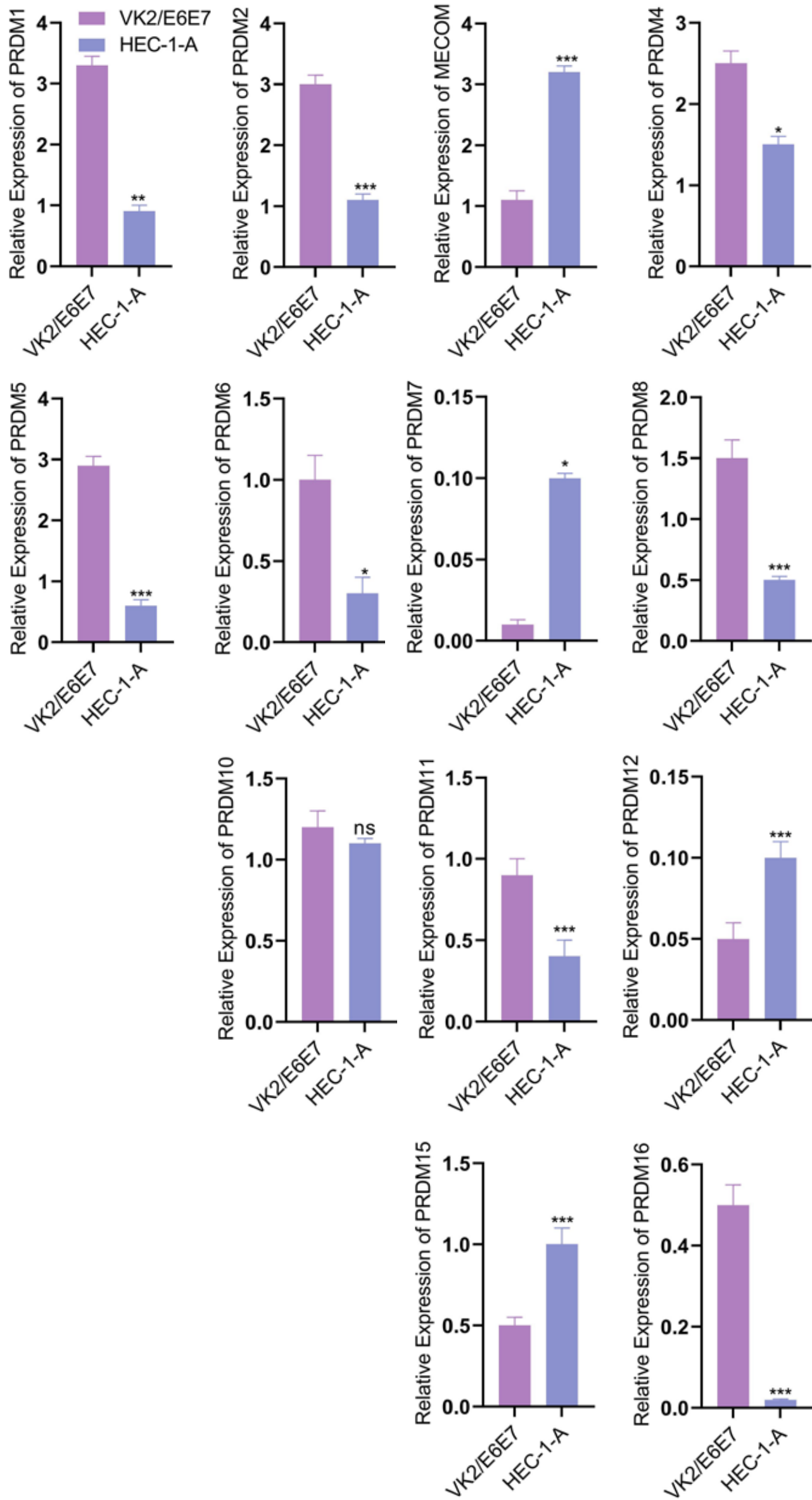

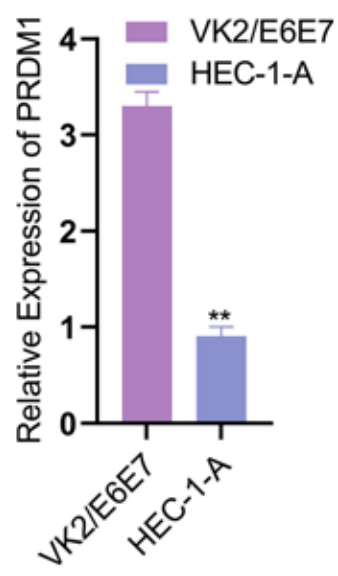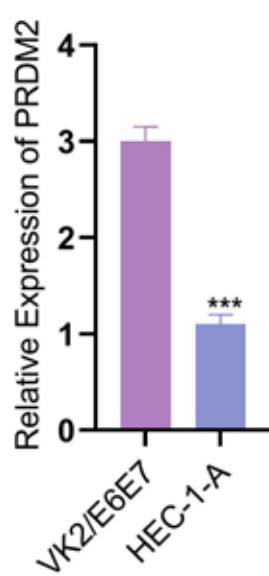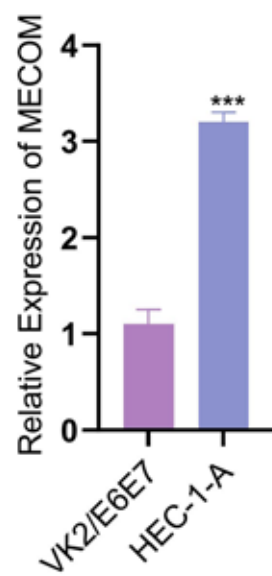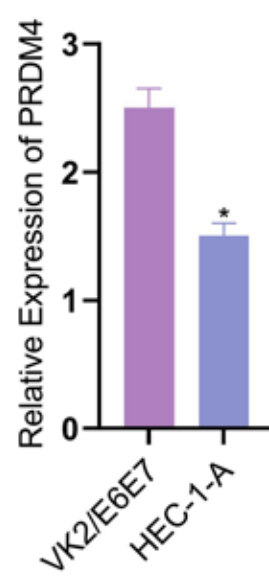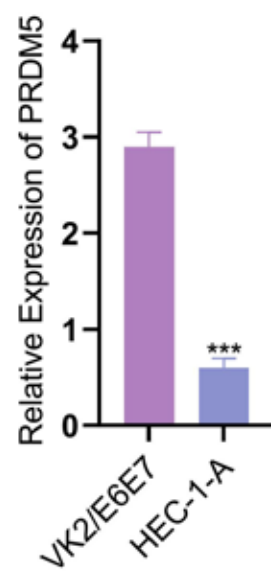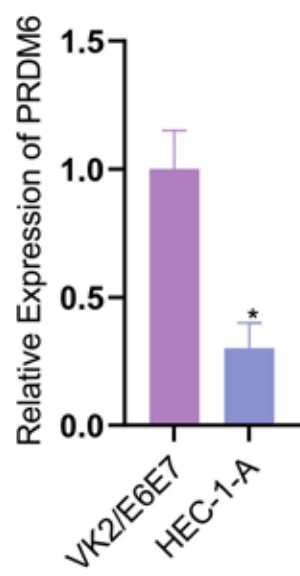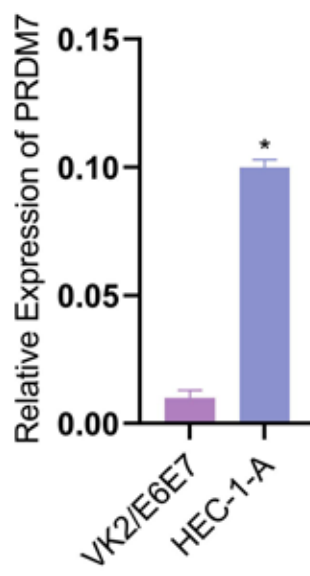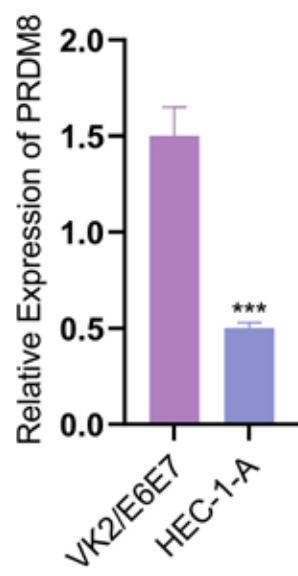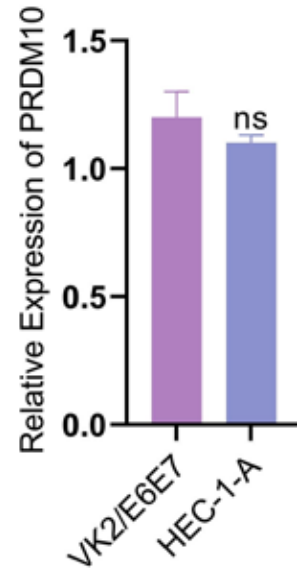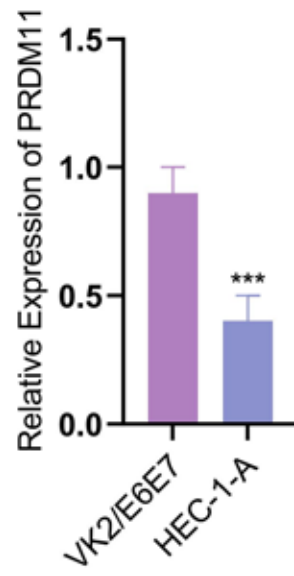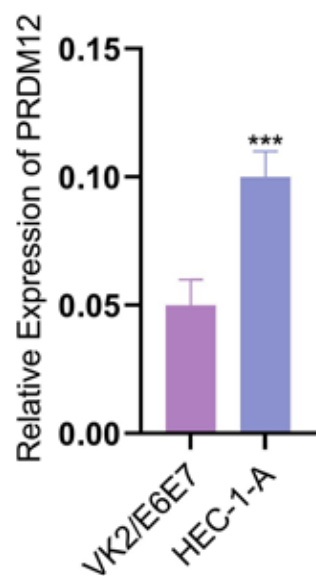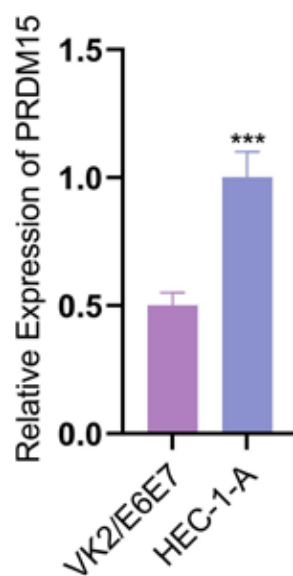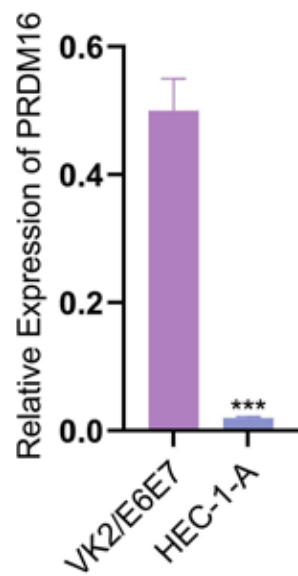

Supplement: Supplementary file 7 — Additional file 7 [file 10020_2024_946_MOESM7_ESM.pdf]
