# Supplementary material for: MECOM and the PRDM gene family in uterine endometrial cancer: bioinformatics and experimental insights into pathogenesis and therapeutic potentials
Source: Mol Med. 2024 Oct 28;30:190. doi: 10.1186/s10020-024-00946-0 (PMC11514642; doi:10.1186/s10020-024-00946-0)
Supplement: Supplementary file 4 — Additional file 4 [file 10020_2024_946_MOESM4_ESM.pdf]

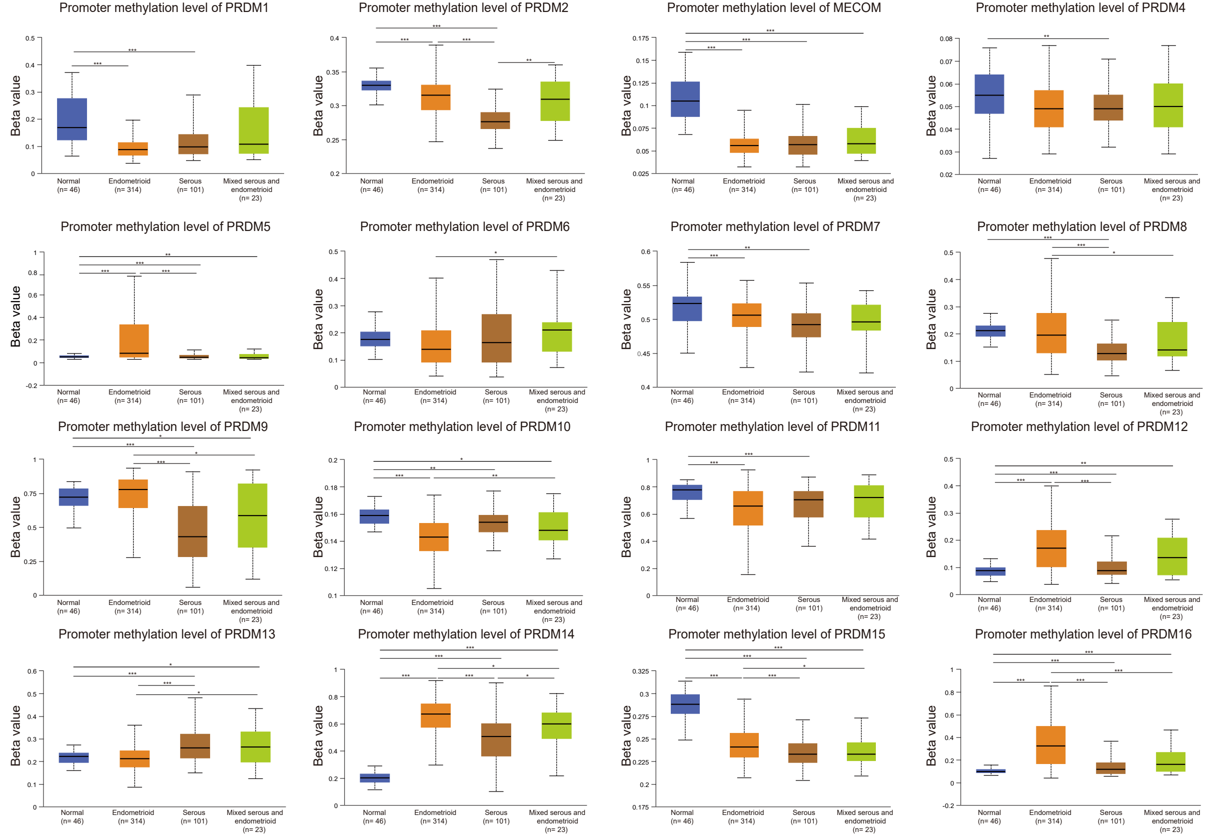
Promoter methylation level of PRDM1
Promoter methylation level of PRDM2
Promoter methylation level of MECOM
Promoter methylation level of PRDM4
Promoter methylation level of PRDM5
Promoter methylation level of PRDM6
Promoter methylation level of PRDM7
Promoter methylation level of PRDM8
Promoter methylation level of PRDM9
Promoter methylation level of PRDM10
Promoter methylation level of PRDM11
Promoter methylation level of PRDM12
Promoter methylation level of PRDM13
Promoter methylation level of PRDM14
Promoter methylation level of PRDM15
Promoter methylation level of PRDM16
Beta value
Normal (n= 46)
Endometrioid (n= 314)
Serous (n= 101)
Mixed serous and endometrioid (n= 23)
