# Supplementary material for: MECOM and the PRDM gene family in uterine endometrial cancer: bioinformatics and experimental insights into pathogenesis and therapeutic potentials
Source: Mol Med. 2024 Oct 28;30:190. doi: 10.1186/s10020-024-00946-0 (PMC11514642; doi:10.1186/s10020-024-00946-0)
Supplement: Supplementary file 5 — Additional file 5 [file 10020_2024_946_MOESM5_ESM.pdf]

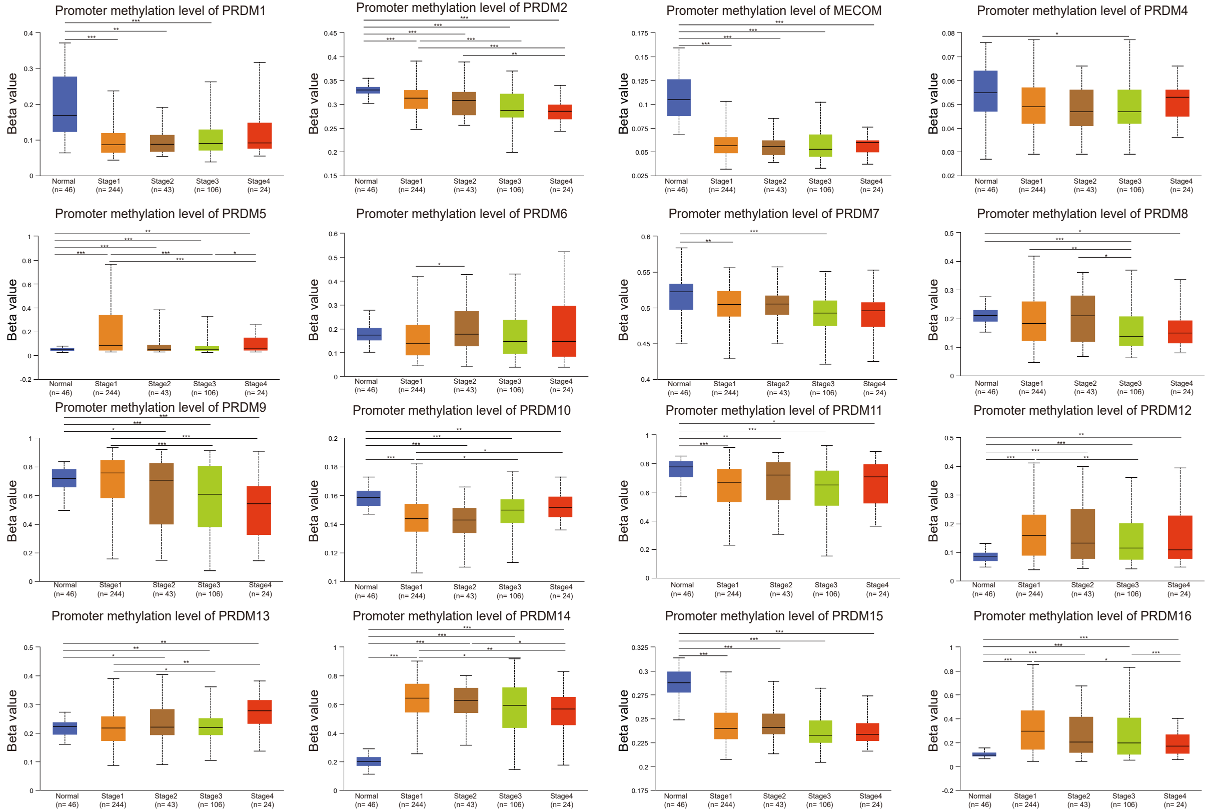
Promoter methylation level of PRDM1
Beta value
0 0.1 0.2 0.3 0.4
Promoter methylation level of PRDM2
Beta value
0.15 0.2 0.25 0.3 0.35 0.4 0.45
Promoter methylation level of MECOM
Beta value
0.025 0.05 0.075 0.1 0.125 0.15 0.175
Promoter methylation level of PRDM4
Beta value
0.02 0.03 0.04 0.05 0.06 0.07 0.08
Promoter methylation level of PRDM5
Beta value
-0.2 0 0.2 0.4 0.6 0.8 1
Promoter methylation level of PRDM6
Beta value
0 0.1 0.2 0.3 0.4 0.5 0.6
Promoter methylation level of PRDM7
Beta value
0.4 0.45 0.5 0.55 0.6
Promoter methylation level of PRDM8
Beta value
0 0.1 0.2 0.3 0.4 0.5
Promoter methylation level of PRDM9
Beta value
0 0.2 0.4 0.6 0.8 1
Promoter methylation level of PRDM10
Beta value
0.1 0.12 0.14 0.16 0.18 0.2
Promoter methylation level of PRDM11
Beta value
0 0.2 0.4 0.6 0.8 1
Promoter methylation level of PRDM12
Beta value
0 0.1 0.2 0.3 0.4 0.5
Promoter methylation level of PRDM13
Beta value
0 0.1 0.2 0.3 0.4 0.5
Promoter methylation level of PRDM14
Beta value
0 0.2 0.4 0.6 0.8 1
Promoter methylation level of PRDM15
Beta value
0.175 0.2 0.225 0.25 0.275 0.3 0.325
Promoter methylation level of PRDM16
Beta value
-0.2 0 0.2 0.4 0.6 0.8 1
Normal (n= 46)
Stage1 (n= 244)
Stage2 (n= 43)
Stage3 (n= 106)
Stage4 (n= 24)
