# Supplementary material for: MECOM and the PRDM gene family in uterine endometrial cancer: bioinformatics and experimental insights into pathogenesis and therapeutic potentials
Source: Mol Med. 2024 Oct 28;30:190. doi: 10.1186/s10020-024-00946-0 (PMC11514642; doi:10.1186/s10020-024-00946-0)
Supplement: Supplementary file 8 — Additional file 8 [file 10020_2024_946_MOESM8_ESM.docx]

**Rebuttal Letter**

Title: MECOM and the PRDM Gene Family in Uterine Endometrial Cancer: Bioinformatics and Experimental Insights into Pathogenesis and Therapeutic Potentials

Reviewer reports:

Reviewer #1: The possibility of MECOM (PRDM3) as a candidate for targeted therapy development endometrial tumors has been studied through descriptive bioinformatic data as well as in-vitro experiment. However, the author's findings did not acceptable based on the result description. Also, it is hard to follow the author's findings and understand the author's conclusions. The authors need to clarify and check figure and manuscript again. Most of finding were from existing database so some major concerns have been raised after carefully reading the work.

**Response**：Thank you for your valuable feedback on our study. We will revise the conclusion section to ensure logical coherence and clarity and check the figures to ensure they accurately reflect the data, along with providing clear legends. While we explored the role of the PRDM family in endometrial cancer (UCEC) through database analysis, our study further focused on the impact of MECOM on the TGF-beta signaling pathway at the cellular level, an area not fully addressed in previous research. Our results suggest that this interaction may play a crucial role in the pathogenesis of UCEC, providing new insights into the role of the PRDM family in cancer biology.

1. What is the full name of PRDM and MECOM? MECOM known as PRDM3.

**Response:** Thank you for your question. We have added the full names of PRDM and MECOM in the introduction section. Specifically: "PRDM stands for PR Domain-Containing Protein, and MECOM stands for MDS1 and EVI1 Complex Locus Protein. The MECOM gene is also known as PRDM3, one of its aliases (PMID: 35174083)."

2. What is the type of endometrial cancer from TCGA, MET500, and CPTAC? More detail sample information needs to be provided by the authors.

**Response:** Thank you for your question. Specific information about the samples can be obtained from the TCGA (https://portal.gdc.cancer.gov), MET500 (https://met500.med.umich.edu/), and CPTAC (http://www.linkedomics.org) websites. We have added a description of the types of endometrial cancer in the Materials and Methods section. Specifically: "The types of endometrial cancer include adenomas and adenocarcinomas, cystic, mucinous and serous neoplasms, and epithelial neoplasms, NOS."

3. It is hard to see the figure 1B.

**Response:** We have enhanced the figure's resolution and ensured that it is more clearly visible in the revised manuscript.

4. What is the expression PRDMs in same sample? Are there any correlation or compensation?

**Response:** Thank you for your suggestion. Although we did not perform an analysis of PRDM expression within individual samples due to database limitations, we did examine the overall correlations between PRDM family members using the available data. We observed significant correlations between certain PRDM genes, such as PRDM2 and PRDM10 (correlation coefficient 0.55) and PRDM4 and PRDM10 (correlation coefficient 0.50), indicating potential compensatory mechanisms. Conversely, negative correlations like those between PRDM2 and PRDM12 (-0.21) and PRDM15 and PRDM12 (-0.20) may also suggest compensatory expression. These observations are reflected in the updated Figure 5C, and the corresponding textual modifications have been made in lines 572-575.

5. How many samples were used for IHC? The definition of "low", "Medium", "High" is not scientific. The quantification data via H-score is required to support the authors' conclusion.

**Response:**Response: Thank you for your question. We have provided a detailed description of the quantification of H-score data in the Materials and Methods section for the Human Protein Atlas (HPA) data analysis. Specifically:"We performed immunohistochemistry (IHC) analysis on 50 UCEC samples. The definitions of 'low,' 'intermediate,' and 'high' expression levels have been replaced by H-score quantification data to provide a scientific basis for our conclusions.Determining intensity levels: Cellular expression intensity was categorized based on staining color depth into different levels, such as no staining (0), weak (1+), moderate (2+), and strong (3+).Estimating the percentage of cells at each intensity level: Tissue sections were observed under a microscope, and the percentage of cells at each intensity level relative to the total number of cells was estimated. Calculating H-score: The H-score was calculated by multiplying the percentage of cells at each intensity level by the corresponding intensity level and then summing the results. The mathematical expression is as follows: H−score=∑i=1n(pi×si)H−score=i=1∑n(pi×si), where pipi is the percentage of cells at intensity level ii (in percent form), sisi is the intensity value for level ii (typically 1, 2, 3, etc.), and nn is the number of intensity levels. The expression was evaluated by the H-score, using the following categorization: H-Score 0 to <100 as low, H-Score 100 to 200 as intermediate, and H-Score >200 to 300 as high." Additionally, we have modified the data in Figure 2B to reflect H-score instead of 'low,' 'intermediate,' and 'high' expressions.

6. It is hard to make a connection between data from UALCAN database and HPA as expression profiling did not match between figure 2A and 2B. The authors need to clarify.

**Response:** Thank you for your question. We have noted the differences between UALCAN and HPA data and have included a more detailed analysis and discussion of these database expression profiles in the revised manuscript. Specifically: "We noticed discrepancies between the data from UALCAN and HPA. UALCAN (http://ualcan.path.uab.edu/) primarily provides large-scale gene expression data for cancer patients and normal populations, including features like differential gene expression and survival analysis. The data come from TCGA and other public cancer genomics databases, focusing on mRNA expression levels. On the other hand, HPA (https://www.proteinatlas.org/) focuses on protein expression levels, utilizing antibody-mediated immunohistochemistry to visualize protein distribution in different human tissues. It also includes RNA expression data and single-cell expression data. Due to UALCAN and HPA focusing on mRNA and protein levels, they may show different expression profiles. mRNA expression levels do not always correlate with protein levels, as post-transcriptional modifications, translation efficiency, and protein degradation can affect the protein levels."

7. It is hard to describe "patients with average weight exhibited lower mRNA expression of MECOM, PRDM7, and PRDM15" because there is no significant difference among group.

**Response:** Thank you for your question. We have revised the description in the results section to: "Additionally, in Figure S3, the mRNA expression of PRDMs in UCEC patients shows a specific correlation with normal endometrial tissue. Compared to normal endometrium, UCEC patients exhibit lower mRNA expression levels of MECOM, PRDM7, and PRDM15, and higher mRNA expression levels of PRDM1, PRDM2, PRDM5, PRDM6, PRDM8, PRDM11, and PRDM16. However, there is no significant difference in the mRNA expression of PRDMs between UCEC patients of different weights."

8. The authors describe "our findings indicate that lower PRDM2, PRDM6, and PRDM11 mRNA expression is associated with favorable survival rates in UCEC patients". However, its expression is down-regulated in figure 1A. The authors need to clarify.

**Response:** Thank you for your question. We have clarified the relationship between the expression levels of PRDM2, PRDM6, and PRDM11 and survival rates in the revised results section of the manuscript, ensuring consistency between the figures and text. Specifically: " Low PRDM2, PRDM6, and PRDM11 mRNA expression in UCEC patients is associated with better survival rates. However, compared to healthy individuals, PRDM2, PRDM6, and PRDM11 mRNA expression is downregulated in UCEC patients. At first glance, this seems paradoxical since PRDMs typically function as tumor suppressor genes in normal cells. However, once cancer has developed, the continuous low expression of PRDMs might no longer promote tumor progression. Instead, it may be associated with a more stable tumor state or less aggressive behavior. The low expression of PRDMs could be linked to enhanced immune surveillance, as PRDM2 expression might affect the immunogenicity of tumor cells. Tumors with low PRDM expression may be more easily recognized and eliminated by the immune system, thereby reducing tumor burden."

9. How can the authors explain methylation status compared to its expression in Figure 1A?

**Response:** We have discussed in detail the methylation status of PRDM genes and their expression levels. In the results section, we included a comparative analysis of how the methylation of PRDM1, PRDM2, and PRDM11 affects gene expression. Specifically: "There is a correlation between the methylation status of PRDM genes and their mRNA expression levels. Methylation is an epigenetic modification, and when a gene's promoter region is highly methylated, transcription initiation is hindered, leading to decreased mRNA expression levels. Studies have shown that the PRDM2, PRDM5, and PRDM16 promoters in lung cancer cells are methylated, resulting in suppressed expression (PMID: 24966940). This study indicates that the methylation status of PRDM gene family members can serve as a regulatory mechanism for gene expression, where increased methylation levels are typically associated with reduced mRNA expression.."

10. How was expression of other PRDMs in cell line? Is it matched with the author's finding in previous figures?

**Response:** We included additional data in Figure S9, showing the expression levels of PRDMs in the normal cervical epithelial cell line VK2/E6E7 and the endometrial carcinoma cell line HEC-1-A. RT-PCR results indicate that the mRNA expression levels of PRDMs in the HEC-1-A endometrial carcinoma cell line are consistent with the results shown in Figure 1A when compared to the normal epithelial cell line. However, the mRNA expression levels of PRDM9, PRDM13, and PRDM14 in UCEC tissues were very low, with almost no detectable signal. We have ensured that these data are clearly presented in the revised manuscript and correspond with the figures and tables.

11. Are there any compensated expression among PRDMs? The authors provide the whole PRDMs expression in individual samples.

**Response:** Thank you for your insightful suggestion. Upon analyzing the available data, we observed some strong positive correlations between certain PRDM family members, such as PRDM2 and PRDM10 (correlation coefficient 0.55), and PRDM4 and PRDM10 (correlation coefficient 0.50). Conversely, we also noted some negative correlations, such as between PRDM2 and PRDM12 (-0.21) and PRDM15 and PRDM12 (-0.20). These negative correlations may suggest a phenomenon of compensatory expression, where the downregulation of one PRDM gene might be counterbalanced by the upregulation of another (Figure 5C). However, due to the limitations of the database, we were unable to obtain a complete dataset to perform a comprehensive analysis across individual samples. We have updated Figure 5C accordingly and made the relevant textual modifications in lines 572-576 to reflect these observations.

Reviewer #2: This study evaluated investigated the PRDM gene family, in particular MECOM's role, in endometrial carcinoma (EC) using bioinformatics analysis. This study proposes some new information about molecular characterization of EC, worth to be further analyzed, with the potential to be also a therapeutic target.

I have the following comments to the Authors:

* Authors did not highlight enough the importance of the problem and why this study was necessary

**Response:** We have revised the introduction to better emphasize the importance of studying MECOM and PRDM family members in UCEC, highlighting the need for new therapeutic targets and a deeper understanding of molecular mechanisms. Specifically, we added the following description: "Despite significant advancements in understanding the molecular mechanisms of tumorigenesis and development (PMID: 30154548; PMID: 32483112) and in targeted molecular therapy research (PMID: 26354523; PMID: 31388127), molecular profiling has been employed for many years to manage these advanced and recurrent UCEC patients. For example, progesterone receptor and estrogen receptor status are used to evaluate whether patients can receive hormone therapy. However, even among some patients with progesterone receptor and estrogen receptor positivity, single-agent aromatase inhibitors only achieve a 10% response rate (PMID: 35397864). Therefore, identifying new targets and further understanding the molecular biology of UCEC remains a pressing priority. These efforts will aid in developing effective targeted chemotherapy strategies in the future. MECOM and PRDM family members play multifaceted roles in cancer. PRDM stands for PR Domain-Containing Protein, and MECOM stands for MDS1 and EVI1 Complex Locus Protein. The MECOM gene is also known as PRDM3, one of its aliases (PMID: 35174083). These proteins influence tumor initiation, progression, and metastasis by regulating gene expression, cell proliferation, differentiation, apoptosis, and signal transduction (PMID: 17942894; PMID: 23048031; PMID: 15077163). The PRDM family affects gene expression through chromatin modifications mediated by their PR domains, which contain the SET domain typical of the protein family (PMID: 36734437). The PRDM genes have been implicated in cell proliferation and differentiation (PMID: 17942894; PMID: 23048031), apoptosis (PMID: 15077163), cell cycle progression (PMID: 15077163), inflammation (PMID: 28698287), metabolism (PMID: 32665551), and the immune microenvironment (PMID: 32290321). Recent studies have suggested that dysregulation of PRDM gene expression in various malignant tumors may be due to genetic factors such as mutations, deletions, and insertions (PMID: 25499759), as well as epigenetic modifications (PMID: 32290321; PMID: 37853459).PRDM5, for instance, has been identified as a transcriptional repressor through its interaction with histone methyltransferase G9a (PMID: 17636019; PMID: 33010785). PRDM14 may play a crucial role in maintaining cellular pluripotency, with its aberrant expression potentially linked to the progression of breast cancer (PMID: 19043588). The EVI1 protein, encoded by the MECOM gene, is a multifunctional transcription factor critical in various hematological malignancies, particularly acute myeloid leukemia (AML). Aberrant expression of EVI1 is associated with poor prognosis, as AML patients with high EVI1 expression typically exhibit a poor response to treatment and shorter survival (PMID: 34958895). Due to their critical roles in cancer, MECOM and PRDM family members are considered potential therapeutic targets. Research directions include developing direct inhibitors of these gene-encoded proteins and exploring their upstream or downstream signaling pathways as intervention points. However, translating these findings into clinical applications requires a deeper understanding of these proteins' functions and the ability to develop specific targeted therapies. Considering the close association between the PRDM gene family and various malignancies, we hypothesize that the PRDM gene family may also be involved in UCEC development and could serve as an effective prognostic biomarker. It could help identify new therapeutic targets for UCEC, ultimately reducing its mortality rate. "

* Authors should add in the introduction a brief revision about what is already known about the study subject

**Response:** We have included a brief review of the current knowledge on MECOM and PRDM family members in the revised introduction, particularly their roles in cancer and potential as therapeutic targets. Specifically:"MECOM and PRDM family members play multifaceted roles in cancer. PRDM stands for PR Domain-Containing Protein, and MECOM stands for MDS1 and EVI1 Complex Locus Protein. The MECOM gene is also known as PRDM3, one of its aliases (PMID: 35174083). These proteins influence tumor initiation, progression, and metastasis by regulating gene expression, cell proliferation, differentiation, apoptosis, and signal transduction (PMID: 17942894; PMID: 23048031; PMID: 15077163). The PRDM family affects gene expression through chromatin modifications mediated by their PR domains, which contain the SET domain typical of the protein family (PMID: 36734437). The PRDM genes have been implicated in cell proliferation and differentiation (PMID: 17942894; PMID: 23048031), apoptosis (PMID: 15077163), cell cycle progression (PMID: 15077163), inflammation (PMID: 28698287), metabolism (PMID: 32665551), and the immune microenvironment (PMID: 32290321). Recent studies have suggested that dysregulation of PRDM gene expression in various malignant tumors may be due to genetic factors such as mutations, deletions, and insertions (PMID: 25499759), as well as epigenetic modifications (PMID: 32290321; PMID: 37853459).PRDM5, for instance, has been identified as a transcriptional repressor through its interaction with histone methyltransferase G9a (PMID: 17636019; PMID: 33010785). PRDM14 may play a crucial role in maintaining cellular pluripotency, with its aberrant expression potentially linked to the progression of breast cancer (PMID: 19043588). The EVI1 protein, encoded by the MECOM gene, is a multifunctional transcription factor critical in various hematological malignancies, particularly acute myeloid leukemia (AML). Aberrant expression of EVI1 is associated with poor prognosis, as AML patients with high EVI1 expression typically exhibit a poor response to treatment and shorter survival (PMID: 34958895).Due to their critical roles in cancer, MECOM and PRDM family members are considered potential therapeutic targets. Research directions include developing direct inhibitors of these gene-encoded proteins and exploring their upstream or downstream signaling pathways as intervention points. However, translating these findings into clinical applications requires a deeper understanding of these proteins' functions and the ability to develop specific targeted therapies. Considering the close association between the PRDM gene family and various malignancies, we hypothesize that the PRDM gene family may also be involved in UCEC development and could serve as an effective prognostic biomarker. It could help identify new therapeutic targets for UCEC, ultimately reducing its mortality rate. ."

* Discussion

* Authors should add in the discussion possible mechanisms explaining their findings

**Response:**We added a possible explanation for our findings in the discussion section, specifically how MECOM might affect the TGF-beta signaling pathway and its impact on UCEC. Specifically: " We hypothesize that MECOM may regulate the transcription levels of Smad proteins by binding to their gene promoter regions or by interacting with Smad proteins to affect their activity, localization, or stability. It, in turn, influences TGF-beta signaling, leading to effects on cell cycle S-phase arrest and apoptosis, thereby impacting endometrial cancer (UCEC). It provides a new mechanistic understanding of MECOM's role in regulating the cell cycle and apoptosis."

* Authors should include in the discussion a comparison of their study with similar studies already present in literature

**Response:** We compared this study with similar studies in the literature, highlighting similarities and differences, and discussed how our findings enhance the current understanding of UCEC. Specifically: " Reports on the role of PRDM in UCEC are limited. However, PRDM family members have been shown to influence various biological behaviors of tumors, such as proliferation, invasion, migration, and apoptosis, either by promoting or inhibiting tumor development in different cancers. The aberrant expression of PRDM family members is associated with disease prognosis in various cancers, suggesting their potential as diagnostic markers and therapeutic targets (PMID: 32290321). Nevertheless, the same PRDM family member may have different roles in different types of cancer, and different PRDM family members may influence tumor development by regulating different genes and signaling pathways, depending on the specific cancer type and microenvironment (PMID: 37853459)."

* In order to make the article more praiseworthy, Authors should include a paragraph analyzing strengths and limitations of their study

**Response:** In the discussion section, we included a paragraph analyzing the strengths and limitations of this study to provide a balanced perspective on our findings. Specifically:"The study provides new insights into the treatment and prognosis assessment of uterine endometrial cancer (UCEC). The expression patterns and methylation status of the PRDM family, particularly MECOM in UCEC, lay the groundwork for identifying new biomarkers and potential therapeutic targets. For example, the upregulation and abnormal methylation of MECOM found in UCEC may hint at its potential value in early disease diagnosis. Moreover, the interaction of MECOM with the TGF-beta signaling pathway reveals new therapeutic mechanisms, potentially aiding in the development of targeted therapies against this pathway. However, discovering the correlation between the expression of PRDM family members and the immune microenvironment in uterine endometrial cancer opens new avenues for designing immunotherapy strategies, especially in personalized medicine and precision treatment. Despite providing new insights into the role of the PRDM family in UCEC, our study has limitations. First, it relies on data from public databases, which may have inconsistencies and quality issues. Second, our conclusions may need validation in a broader patient population due to the lack of large-scale clinical samples and prospective studies. We have not yet determined how MECOM influences the TGF-beta signaling pathway and UCEC through its effects on Smad proteins. In future experiments, we can first verify how MECOM affects Smad proteins. It can be done by using RNA-seq or microarray analysis to examine the expression profiles of Smad-related genes under different levels of MECOM expression, identifying potential downstream targets.Furthermore, we can establish xenograft tumor models in nude mice to evaluate the impact of MECOM on UCEC growth. While we explored the function of MECOM at the cellular level, these findings need further confirmation through in vivo experiments and clinical trials. Therefore, while our study offers valuable preliminary insights, more research is needed before these findings can be translated into clinical applications."

* In order to stimulate research of this topic, Authors may include in the discussion possible research ideas based on their observations

**Response:** Based on our observations, we propose a potential research direction to stimulate further studies on the roles of MECOM and PRDM family members in UCEC. Specifically: "We have not yet determined how MECOM influences the TGF-beta signaling pathway and UCEC through its effects on Smad proteins. In future experiments, we can first verify how MECOM affects Smad proteins. It can be done by using RNA-seq or microarray analysis to examine the expression profiles of Smad-related genes under different levels of MECOM expression, identifying potential downstream targets.Furthermore, we can establish xenograft tumor models in nude mice to evaluate the impact of MECOM on UCEC growth."

* The ESTRO/ESGO/ESP guidelines for the management of EC proposed a novel risk stratification model including molecular TCGA molecular groups to assess the prognosis of EC in association with classic, well-known, clinicopathologic prognostic factors of EC (such as myometrial invasion, histotype or lymph vascular space invasion). Some recent studies analyzed the relationship between TCGA groups and classic prognostic factors (grade, myometrial invasion, LVSI) and I believe the discussion should include those recent findings in order to add also a clinical point of view to the article (e.g. PMID: 34088515; PMID: 35078650).

**Response:** Response: We have incorporated the latest findings from the ESTRO/ESGO/ESP guidelines and the relationship between TCGA molecular subtypes and traditional prognostic factors into the discussion, integrating a clinical perspective. Specifically: "The TCGA project, through extensive sequencing and analysis of numerous cancer samples, has unveiled the molecular landscape of various cancers, including UCEC. In UCEC, TCGA identified several key molecular subtypes: POLE-ultramutated, microsatellite instability (MSI), p53-mutant, and no specific molecular profile (NSMP). These subtypes reflect the genetic heterogeneity of tumors and are closely associated with clinical phenotypes, treatment responses, and prognosis. From a clinical perspective, integrating TCGA molecular subtyping with traditional prognostic factors (such as age, tumor grade, stage, lymph node status, etc.) can provide more precise patient risk stratification. For instance, a young patient may have a better overall physiological condition, but if her tumor is of the p53-mutant subtype, her prognosis may be worse than expected, necessitating a more aggressive treatment approach. Conversely, an older patient, even with a tumor classified as POLE-ultramutated, may have a better survival outlook due to favorable molecular characteristics despite facing higher risks from surgery and chemotherapy. Moreover, as mentioned in updated guidelines, molecular biomarker testing can aid clinicians in making treatment decisions based on specific molecular features of the patient's tumor, opting for the most appropriate treatment regimen, such as targeted therapy or immunotherapy, rather than solely relying on traditional clinicopathological indicators. In summary, combining the latest molecular biology discoveries with clinical practice can enhance our understanding and treatment of endometrial cancer, offering more personalized medical care for patients (PMID: 34088515; PMID: 35078650).”

Comments from Editor to authors

Authors should check Figure 1 PRDM14, PRDM9, PRDM13 bar graph looks strange. Authors should elaborate more about schematic diagram in figure legend.

**Response:** Thank you for reviewing our work and providing valuable feedback. Regarding the bar graphs for PRDM14, PRDM9, and PRDM13 in Figure 1, we confirm that the mRNA expression levels of these genes in UCEC tissues are indeed very low, with almost no detectable signal, which results in the lower values presented in the bar graphs. These data have been verified through multiple experimental repetitions, and the original data also show consistent results. We believe that these low expression levels reflect the relatively weak or insignificant biological role of these genes in UCEC.

Additionally, we have revised the figure legend according to your suggestion, providing more detailed explanations of the schematic diagram, including the specific content represented by each bar graph. We hope this revision will help clarify the biological significance of the data presented.
